# APOE Genotype Differentially Modulates Prion Pathology in a Mouse Model

**DOI:** 10.21203/rs.3.rs-7820890/v1

**Published:** 2025-10-30

**Authors:** Anita M. Lizińczyk, Joanna E. Pankiewicz, William L. Cullina, Leor A. Franco, Patrick M. Sullivan, Martin J. Sadowski

**Affiliations:** New York University Grossman School of Medicine; New York University Grossman School of Medicine; New York University Grossman School of Medicine; New York University Grossman School of Medicine; Duke University School of Medicine; New York University Grossman School of Medicine

**Keywords:** Alzheimer’s disease, apolipoprotein E, astrocytes, microglia, neurodegeneration, neuroinflammation, prion diseases, prion protein

## Abstract

*APOE* polymorphism affects the risk of occurrence and the rate of progression in several neurodegenerative diseases including Alzheimer’s disease, primary tauopathies, α-synucleinopathy, and age-related macular degeneration, but its role in prionoses remains unestablished. Using *APOE* targeted replacement (TR) mice, we investigated how *APOE* genotype affects key neurodegenerative mechanisms involved in prion pathology. Male and female *ε2/ε2*, *ε3/ε3*, and *ε4/ε4 APOE*-TR mice were inoculated with 22L mouse-adapted scrapie strain or normal brain homogenate and monitored with behavioral testing from 10-week post inoculation (wpi.) onward. Mice were euthanized at 23 wpi. when all prion-infected animals were symptomatic, and their brains were analyzed for multiple neuropathological, biochemical, and transcriptomic metrics. *ε4/ε4*_22L_ mice featured the shortest disease latency time, the worst neurological score, and the highest load of spongiform lesions. *ε2/ε2*_22L_ mice performed significantly better than *ε4/ε4*_22L_ mice but significantly worse than *ε3/ε3*_22L_ animals. Numerous aspects of PrP proteinopathy were exacerbated in the presence of the *ε4* allele including increased PrP^Sc^ accumulation, reduced PrP solubility, and increased PrP oligomerization. These metrics were comparable between *ε2/ε2*_22L_ and *ε3/ε3*_22L_ mice. Prion pathology significantly increased brain apolipoprotein (apo) E levels, with the greatest increase in *ε4/ε4*_22L_ mice. All apoE isoforms formed complexes with conformationally altered PrP, but this interaction was the strongest in *ε4/ε4*_22L_ mice. *ε4/ε4*_22L_ mice had the highest load of reactive microglia and astrocytes and upregulation of transcriptomic markers typical of neurodegenerative microglia and astrocytes, followed by *ε2/ε2*_22L_, with *ε3/ε3*_22L_ having the lowest. Thus, *APOE* polymorphism differentially regulates the progression of prion pathology attributable to two *ε4*-affected mechanisms: increased conversion and accumulation of PrP^Sc^ and worsened prion-associated neuroinflammation. Though less severely than *ε4*, the *ε2* allele also increased the inflammatory response, rendering disease outcome worse relative to the *ε3* allele. Our findings suggest both *ε4* and *ε2* alleles are disadvantageous determinants in prion pathology.

## Introduction

Apolipoprotein (apo) E is a 34-kDa lipid transporting protein encoded by the *APOE* gene located on chromosome 19q13.32 and expressed by hepatocytes, astrocytes, immune cells of the myeloid-lineage, vascular smooth muscle cells and adipocytes [87]. *APOE* polymorphism includes three common alleles *ε2, ε3*, and *ε4*, with world-wide distribution frequencies of 6.4%, 78.3%, and 14.5%, respectively [35]. They encode respective isoforms of the apoE protein, which differ in the presence of cysteine and arginine at positions 112 and 158 and feature impactful dissimilarities in tertiary structure, lipid binding ability, and receptor-mediated clearance [22, 70]. *APOE* polymorphism influences the risk of occurrence and the rate of progression in several cardiovascular and neurodegenerative diseases [5, 119]. The presence of the *ε4* allele significantly increases risk of coronary artery disease, while the *ε2* allele is associated with elevated plasma triglyceride level, and increases risk of peripheral vascular disease and carotid atherosclerosis [32, 101]. Since its discovery in the early 90’s, *APOE* polymorphism has remained the strongest identified genetic factor affecting risk of late-onset Alzheimer’s disease (LOAD) with the *ε4* allele being associated with increased disease occurrence [25, 26, 119], while the *ε2* allele effectively lowering the risk among *ε4* allele non-carriers [24, 88]. This strong clinical effect is canonically explained by the opposing effects of ε*4* and *ε2* alleles on brain clearance of soluble β-amyloid (Aβ) peptide [20], Aβ aggregation [37], and Aβ plaque formation [75, 97], which are critical steps in establishing early AD pathology. Irrespective of promoting LOAD’s occurrence, the *ε4* allele is associated with worse outcome of once established disease, via modulation of several disease mechanisms, including spread of tau pathology [99], neuroinflammatory response [98], and endo-lysosomal system dysfunction [100], which all are potent contributors to faster rate of dementia progression among *ε4* carriers compared to non-carriers [21, 27, 86]. Besides LOAD, *APOE* polymorphism also affects the risk of occurrence and the rate of progression of several other neurodegenerative diseases including primary tauopathies, α-synucleinopathies, and age-related macular degeneration, but unlike LOAD, in some of these entities not the *ε4* allele but the *ε2* allele has been found to produce worse outcomes [46, 58, 128].

Prion diseases or prionoses are neurodegenerative proteinopathies, characterized by the accumulation of disease specific scrapieform conformer (PrP^Sc^), which sets off a neurodegenerative cascade including proinflammatory activation of microglia and astrocytes [3, 4, 15, 18, 42, 77] leading to widespread synaptic and neuronal loss and an ultimately fatal outcome [71, 85, 91]. PrP^Sc^ arises from the cellular prion protein (PrP^C^) in a process known as recycling propagation, in which PrP^Sc^ binds PrP^C^ during its recycling cycle between the plasma membrane and the endosomal compartment and forces PrP^C^ to adopt its own β-sheet-rich secondary conformation [40, 41, 48]. Transition between PrP^C^ and PrP^Sc^ conformers is gradual and associated with a number of physicochemical changes, including reduced detergent solubility, oligomerization, and acquisition of partial proteolytic resistance, which is a hallmark property of PrP^Sc^ [30, 40, 41, 78, 81, 115]. Prionoses affect both human and non-human mammalian species [2, 91]. The list of human prionoses include Creutzfeldt-Jakob disease (CJD), Gerstmann Straussler Scheinker syndrome, fatal familial insomnia, variably protease-sensitive prionopathy and kuru. Sporadic CJD (sCJD) with an annual incidence approximated at one case per million is the most common of the human prionoses [38, 67, 91]. Given several shared characteristics between sCJD and other neurodegenerative diseases, which include strong association with aging, misfolded protein centered pathology, and chronic neuroinflammatory activation, several past studies have explored how *APOE* polymorphism might influence the risk of sCJD occurrence. While some initial reports suggested a modest uptick in incidence among *ε4* carriers [6, 118] this association was refuted by subsequent studies [72, 95, 126]. Expanding knowledge on the modulatory effects played by *APOE* alleles in various mechanisms of neurodegeneration and separating these mechanisms into those regulating the risk of disease occurrence from those affecting the rate of progression [119] merit a careful examination whether *APOE* polymorphism might influence the course of neurodegeneration in prionoses. However, the limited number of clinical cases and well-recognized complexity of human prion disease, which includes factors like codon 129 polymorphism, PrP^Sc^ subtypes, and variable presence of Aβ co-pathology [33, 79], render systematic analysis of the *APOE* polymorphism effect on disease progression in human prion entities arduous to conduct and inherently underpowered [126]. To control for these variables we used *APOE* targeted replacement (*APOE*-TR) mice, where both murine *Apoe* alleles are replaced by human *APOE* alleles [110], and infected them with 22L mouse adapted scrapie strain, which is an established and reliable laboratory model of prion disease featuring limited variability in disease latency and neuropathological metrics [8, 9, 77, 93]. Using this model, we determined the detrimental effect of the *ε4* allele and to a lesser extent the *ε2* allele on progression of neurodegeneration compared to the *ε3* allele.

## Material and methods

### Material and reagents

Unless otherwise specified, all reagents and chemicals were purchased from Sigma-Aldrich (St. Louis, MO). Information on primary and secondary antibodies used for Western immunoblotting and immunohistochemistry is provided in Tables 1 and 2, respectively. Sequences of primers used for Reverse Transcription Quantitative Polymerase Chain Reaction (RT-qPCR) are listed in Table 3. All primers were made to order by Sigma-Aldrich.

### Transgenic animals

All mouse care and experimental procedures were approved by Institutional Animal Care and Use Committees of the New York University Grossman School of Medicine. *Apoe*^*−/−*^, *ε2/ε2*, *ε3/ε3*, and *ε4/ε4* strains have been detailed in previous publications [51, 127]. *ε2/ε2*, *ε3/ε3*, and *ε4/ε4* lines are *APOE* targeted replacement mice, where both murine *Apoe* alleles are replaced by isogenic human *APOE* alleles and those remain expressed under the endogenous *Apoe* promoter. We maintain a colony of *APP/PS1/Apoe*^*−/−*^, *APP/PS1/ε2/ε2*, *APP/PS1/ε3/ε3*, and *APP/PS1/ε4/ε4* mice, which are heterozygous for the *APP/PS1* transgene [56, 75]. For this project we used non-transgenic offsprings from this colony, which did not carry the *APP/PS1* transgene. All animals were subjected to genomic DNA analysis. They genotyped negatively for the *APP/PS1* transgene, while their *APOE* genotype was confirmed by restricted fragment length polymorphism of the *APOE* amplification product as previously described [56]. All mice used in this study were on C57BL/6 background.

### Experimental design

Prion disease was induced by intraperitoneal inoculation with 22L mouse adapted scrapie strain following our published protocols [77, 93]. Control animals were intraperitoneally inoculated with the normal brain homogenate (NBH). Mice were inoculated at the age of 10–12 weeks, maintaining ~ 50%:50% female: male ratio per each experimental group. Mice were euthanized at 23 weeks post inoculation (wpi.), when all 22L-inoculated groups displayed overt neurological signs of prion disease, while NBH inoculated control mice appeared healthy. A subset of 22L-inoculated mice also was euthanized at 15 wpi. when the mice remain presymptomatic, to assess accumulation of PrP^Sc^ in the lymphoreticular system (LRS) and characterize early stage of neuroinvasion. In the manuscript, 22L-inoculated mice euthanized at 15 and 23 wpi. are alternatively referred to as presymptomatic and symptomatic animals, respectively.

### Prion inoculation, animals’ care, and behavioral testing

The 22L prion inoculum was prepared from the brains of C57BL/6 mice, which were infected with 22L mouse adapted scrapie strain and housed in an Animal Biosafety Level 2 facility until they reached the terminal stage of prion disease. Their brains were harvested and homogenized under sterile conditions in the tissue homogenization buffer (THB) maintaining 1:10 weight to volume ratio. The THB consists of 20 mM Tris-HCL pH 7.4, 250 mM sucrose, 1 mM ethylenediaminetetraacetic acid, 1 mM egtazic acid and 10 μg/mL of Complete Proteinase Inhibitor Cocktail (cOmplete) (Roche Life Science, Indianapolis, IN). After preparation, the inoculum was immediately aliquoted, flash-frozen, and stored at −80°C until use. NBH was prepared from the brains of healthy C57BL/6 mice following the same protocol. A single prepared batch of 22L inoculum and NBH was used for the entire study. For the inoculation, aliquots of the 22L inoculum or NBH were taken out from the cryostorage and thawed. Each animal received a single intraperitoneal injection containing 100 μL of either 22L inoculum or NBH. Remnants of the aliquots were never reused but neutralized with an excess of sodium hypochlorite and disposed.

Following the inoculation mice were kept in a pathogen-free Animal Biosafety Level 2 facility with 12/12-hour light/dark cycle and *ad libitum* food and water access. Their general health and well-being were assessed twice a week following established standards of good husbandry practice [12]. From 10 wpi. onward, mice were evaluated weekly for the first signs of prion disease using a parallel bar crossing test, which was carried out by two independent examiners blinded to *APOE* genotype and inoculum type. This testing evaluates an animal’s competency to cross a series of parallel bars that are 3 mm in diameter and set 7 mm apart. An animal displaying difficulties in initiating and/or completing this task in a timely and coordinated manner for three weeks in a row is considered clinically symptomatic and the first week that the positive score is assigned is considered the onset of clinical disease. Severity of neurological symptoms were longitudinally characterized using the Total Scrapie Score (TSS), which is an equally weighted composite of the following scorable metrics: somnolence, hind limb weakness, kyphosis, walk, and body condition. These behavioral metrics are scored based on the following criteria: 0 = normal, 1 = subtle, 1.5 = mild, 2 = moderate, 2.5 = advanced, and 3 = severe [16, 17, 77, 104]. Their sum constitutes the TSS, which ranges from 0 in healthy animals to 15 points in terminally sick ones. TSS was assessed on a weekly basis starting from the 100th day post-inoculation (dpi) by two independent examiners who remained blinded to the animal *APOE* genotype.

### Animal euthanasia and tissue harvesting

At the conclusion of the experiment animals were euthanized by a single intraperitoneal injection of Euthasol (500 μl/kg) (Virbac AH, Inc.; Westlake, TX). Once they showed absence of pain and corneal responses, they were transcardially perfused with heparinized, ice-cold 10 mM phosphate-buffered saline (PBS) pH 7.4. Their brains were extracted from the skulls and carefully stripped from the dura and vessels under the AmScope stereoscopic microscope (AmScope, Chino, CA). The olfactory bulbs, the brain stem, and the cerebellum were removed, and the corpus callosum was dissected to separate the hemispheres. The cortical mantle including the hippocampus was dissected out from the left hemisphere and either flash-frozen and stored at −80°C or immediately used for RNA extraction. The total RNA was extracted using RNeasy Mini Kit (Qiagen Sciences Inc., Germantown, MD) following the manufacturer-provided protocol. The resulting extract was treated with 2 U of DNAse I per brain (Qiagen Sciences Inc.), flash frozen and stored at −80°C for transcriptomic analysis. The right brain hemisphere was cut in the frontal plane at ~ 1 mm anterior to the bregma. The rostral part was immersion-fixed in 2% phosphate-buffered formalin and embedded in paraffin. The caudal part was immersion fixed in 2% paraformaldehyde in 0.1 M phosphate buffer (PB), pH 7.4 at 4°C for a week and then dehydrated in a solution of 2% dimethyl sulfoxide and 20% glycerol in 0.1 M PB, pH 7.4 at 4°C until sectioning.

### Western immunoblot analyses

Brain homogenate samples were removed from cryostorage, thawed, weighted, and homogenized in the THB maintaining 1:10 tissue weight to the THB volume ratio. A three-step homogenization protocol was followed where the tissue was first manually fragmented by grinding with a pestle, then triturated by repeated passing through a 28-gauge needle and finally sonicated. The remaining cellular fragments were cleared by centrifugation at 10,000 × g and 4°C for 3 min. The protein concentration in the resulting supernatant was measured by bicinchoninic acid (BCA) method using Pierce^™^ Micro BCA Protein Assay Kit (Thermo Fisher Scientific, Waltham, MA) according to the manufacturer’s protocol. Samples containing 5 μg of the total protein were resolved by sodium dodecyl sulfate-polyacrylamide gel electrophoresis (SDS-PAGE) under reducing conditions using 10% gels. Resolved protein was transferred onto nitrocellulose membranes, which were blocked overnight in 5% non-fat milk at 4°C and then incubated with primary and then horse radish peroxidase-conjugated secondary antibodies listed in Table 1. The membranes were treated with SuperSignal West Pico PLUS Chemiluminescent Substrate (ThermoFisher Scientific) and apposed to HyBlot CL^®^ autoradiography films (Thomas Scientific, LLC, Swedesboro, NJ), which then were developed. For immunoblotting of the apoE protein the Western blot protocol was modified by increasing the amount of the total protein in electrophoresed samples to 20 μg and using 5% soy milk for the overnight block. To confirm equal protein load, the nitrocellulose membranes were stripped with Restore^™^ Western Blot Stripping Buffer (Thermo Fisher Scientific) and immunoblotted against β-actin.

Autoradiography films were digitized at the resolution of 600 dots per inch and saved in TIFF format. Protein band optical densities (OD) were quantified using NIH ImageJ v2.1.0/1.53c (Bethesda, MD) following our previously established protocols [7, 77, 78]. For PrP protein analysis, OD of its three bands was totaled.

### PrP detection

Aliquots of brain homogenate containing 10 μg of total protein were diluted with 10 mM PBS to the final protein concentration of 1 μg/μL and treated with Proteinase K (PK) (Roche Life Science) at 37°C for 45 min. maintaining 10:1 protein to enzyme weight ratio. PK activity was quenched by adding 4 μL of 100 mM phenylmethylsulfonyl fluoride (PMSF) per sample and placing the samples in ice bath for 5 min. Samples were centrifuged at 20,000 × g and 4°C for 45 min. Resulting pellets were resuspended in 20 μL of 10 mM PBS and 20 μl of sample buffer containing β-mercaptoethanol, boiled, and resolved on 12.5% SDS-PAGE. Following Western blot transfer, the PrP protein was immunodetected and densitometrically quantified as described above.

To detect the presence of PrP^Sc^ in the LRS we homogenized spleen tissue, which was first cut up into small pieces using a thin surgical blade and then thoroughly sonicated in Dulbecco’s phosphate buffered saline (DPBS) without Ca^2+^ and Mg^2+^ and supplemented with 10 μg/mL cOmplete. Remnants of unhomogenized tissue were cleared by centrifugation at 10,000 × g and 4°C for 3 min. and the protein concentration in the resulting supernatant was measured by the BCA method. Samples containing 500 μg of total protein were diluted with DPBS to obtain the final volume of 100 μL, mixed with equal volume of 4% Sarkosyl in DPBS, and incubated at 37°C for 10 min. with constant agitation in a ThermoMixer^®^ C (Eppendorf North America, Enfield, CT). Then, Benzon nuclease and MgCl_2_ were added to the final concentrations of 50 U/mL and 1 mmol/L, respectively, and the samples were incubated again at 37°C for 30 min. To enhance sensitivity of PrP^Sc^ detection, the total protein in the sample was precipitated with sodium phosphotungustic acid (NaPTA) [93, 94]. A 4% stock solution of NaPTA was prepared in 170 mmol/L MgCl_2_ and added to achieve the final NaPTA concentration of 0.3% in the sample. Samples were incubated at 37°C for 30 min. in a ThermoMixer^®^ C and then centrifuged at 15,800 × g and 4°C for 30 min. Resulting pellets were resuspended in 50 μL of 0.1% Sarkosyl in DPBS and sonicated for 30 sec. [93, 120]. Ten μL of sarkosyl solubilized pellet was mixed with 38 μL of PBS and digested by adding 2 μL of 1 μg/μL PK solution at 37°C for 45 min. PK activity was quenched by adding 6 μL of 100 mM PMSF per sample and placing the samples in ice bath for 5 min. PK-digested samples were centrifugated at 20,000 × g and 4°C for 45 min. Resulting supernatant was discarded while the pellets were resuspended in 20 μL of 10 mM PBS and 20 μL of sample buffer containing β-mercaptoethanol, boiled, and resolved on 12.5% SDS-PAGE. Following Western blot transfer, the PrP protein was immunodetected and densitometrically quantified as described above.

### PrP detergent solubility assay

Fifty microliters of brain homogenate were mixed with 50 μL THB containing 1% Triton X-100 and 1% sodium deoxycholate and incubated on ice for 60 min. The samples were sonicated for 30 sec., incubated at 37°C for 60 min., and centrifuged at 10,000 × g at 4°C for 3 min. Protein concentration in the supernatant was assayed using the BCA method. Aliquots containing 100 μg of protein were diluted with 10 mM PBS to achieve 1μg/μL protein concentration and subjected to ultracentrifugation at 150,000 × g and 4°C for 60 min. using the TLA120.2 fixed-angle rotor in Optima TL ultracentrifuge (Beckman Coulter, Indianapolis, IN). Ultracentrifugated supernatant containing detergent soluble PrP fraction was transferred into new tubes, while the pellets containing detergent insoluble PrP fraction were solubilized by sonication in 100 μL of THB containing 0.5% Triton X-100 and 0.5% sodium deoxycholate. Ten microliters of the supernatant or 10 μL of the solubilized pellet were mixed with an equal volume of sample buffer containing β-mercaptoethanol, boiled, and resolved on 12.5% SDS-PAGE. Following Western blot transfer, the PrP protein was immunodetected and densitometrically quantified as described above.

### Characterization of PrP oligomers

Brain homogenate samples containing 600 μg of total protein were diluted with 10 mM PBS to the final volume of 200 μL, mixed with 20 μL of 10% Sarkosyl and incubated on ice for 30 min. Then they were loaded on the top of the sucrose density gradient, which was formed in polyallomer centrifuge tubes by carefully layering 300 μL of 60%, 50%, 40%, 30%, 20% and 10% sucrose solution prepared in deionized water. Velocity sedimentation was performed using TLS-55 swinging-bucket rotor in an Optima TL ultracentrifuge (Beckman Coulter, Indianapolis, IN) at 200,000 × g and 4°C for 90 min. Fourteen fractions (145 μL each) were collected from the top to the bottom of each ultracentrifugated sample. Twenty microliters from each fraction were mixed with an equal volume of sample buffer containing β-mercaptoethanol, boiled, and resolved on 12.5% SDS-PAGE. Following Western blot transfer, the PrP protein was immunodetected and densitometrically quantified as described above. OD of the PrP signal in each fraction was converted to percentage value using the sum of OD values in all 14 fractions as denominator. Bovine serum albumin (molecular weight 68 kDa), alcohol dehydrogenase (150 kDa) and apoferritin (443 kDa) were used as molecular weight markers. They were subjected to the same velocity sedimentation, SDS-PAGE, and Western blot protocols as PrP oligomers and detected with InstantBlue Coomassie Protein Stain (ThermoFisher Scientific).

### Immunoprecipitation and characterization of PrP/apoE complexes

M-280 Sheep anti-mouse IgG magnetic Dyanabeads^™^ (Thermo Fisher Scientific) were coated with anti-human apoE monoclonal antibody (mAb) HJ15.3 [44, 60, 103]. For each immunoprecipitated brain homogenate sample a 50 μL of manufacturer provided bead solution was mixed with 15 μg of the antibody and incubated in room temperature for 3 hrs. HJ15.3 coated beads were added to samples of brain homogenate containing 400 μg of total protein in 400 μL volume and incubated overnight at 4°C with constant mixing on a Roto-Bot programmable rotator (Benchmark Scientific, Sayreville, NJ). On the following day, the beads were magnetically separated, washed with 10 mM PBS pH 7.4, and incubated in a solution containing 0.05 M Tris-HCL pH 8.0, 0.15 M NaCl and 2% Sarkosyl for 30 min. in room temperature with constant mixing to remove nonspecifically bound brain proteins. This step was followed by additional 30-min. and 5-min. incubations in 0.05 M Tris-HCl solution pH 8.0 containing 0.5 M NaCl and 1% Sarkosyl in room temperature, with constant mixing. Finally, the beads were magnetically separated and resuspended in 20 μL of 10 mM PBS and 20 μL of sample buffer containing β-mercaptoethanol, boiled, and resolved on 12.5% SDS-PAGE. Following Western blot transfer, the PrP protein was immunodetected as described above. To confirm the presence of the apoE protein in immunoprecipitated complexes, the nitrocellulose membranes were stripped with Restore^™^ Western Blot Stripping Buffer (Thermo Fisher Scientific) and immunoblotted with anti-human ApoE goat polyclonal antibody (Table 1).

### Histology, immunochemistry, and quantitative neuropathology

Paraffin blocks containing the rostral portion of the right hemisphere were cut into 5-μm-thick coronal sections, which were then stained with hematoxylin-eosin. The load of spongiform lesions in the M1 primary motor cortex was quantified at three approximated bregma levels (+ 1.0 mm, + 1.2 mm, and + 1.4 mm) following our previously published protocols [77]]. The caudal portion of the right hemisphere was cut serially using a freezing microtome (Leica Microsystems, Weltzer, Germany) into 40-μm-thick coronal sections, which were alternately collected into 10 series and stored in a cryoprotectant solution consisting of 30% ethylene glycol and 30% sucrose in 0.1M PB, pH 7.4. Randomly selected series of sections were immunostained against the following antigens [1] cluster of differentiation (CD) 230 (a.k.a. prion protein [PrP]), [2] ionized calcium adaptor protein 1 (IBA1), [3] cluster of differentiation (CD) 68, [4] glial fibrillary acidic protein (GFAP), and [5] complement component 3 (C3) in combination with GFAP. An antigen retrieval protocol was used for all immunostainings and involved incubating the sections in 10 mM sodium citrate pH 6.0 with 0.05% Tween 20 at 85°C for 15 min. For anti-CD230 immunostaining, sections were additionally incubated in 98% formic acid at room temperature for 10 min. to disrupt β-sheet-pleated secondary structure of the PrP^Sc^ conformer. Non-specific staining was reduced using a blocking mixture which contained 10% normal goat serum, 1% bovine serum albumin and 0.3% Triton X-100 in 10 mM PBS pH 7.4 in room temperature for two hours. For mouse-derived primary antibodies, the mouse-on-mouse blocking reagent (Vector Laboratories; Burlingame, CA) was added to the blocking mixture at the amount of 1.5 μL per 1mL. The list of primary antibodies and fluorochrome-conjugated secondary antibodies, along with their working dilutions is provided in Table 2. Double anti-GFAP/anti-C3 immunostaining was performed using a mixture of primary antibodies to respective antigens, followed by a mixture of fluorochrome-conjugated secondary antibodies. All immunostainings were carried out on free floating sections. Sections were washed thrice with excess 10 mM PBS pH 7.4 and 0.1% Triton X-100 between each step of the protocol. Immunostained sections were carried onto glass histological slides, briefly air-dried, and coverslipped using Depex mounting medium (Thermo Fisher Scientific, Waltham, MA). They were digitized and subjected to quantitative analysis following our published protocols [75–77]. Quantitative metrics included [1] integrated density (ID) of anti-CD230 (PrP) immunostaining, the load of [2] IBA1^+^ and [3] CD68^+^ microglia, [4] the load of GFAP^+^ astrocytes, and [5] ratio of C3^+^ to GFAP^+^ immunostaining in astrocytes. All quantitative analyses were performed in the S1 primary somatosensory cortex at three approximated bregma levels (0.0 mm, −0.4 mm, and − 0.8 mm).

### NanoStringTM nCounter^®^ analysis of glial transcript

Aliquots of previously isolated total RNA were removed from − 80°C cryostorage and assayed for purity and integrity using a 2100 Bioanalyzer (Agilent Technologies Inc., Santa Clara, CA). Only samples with the RNA Integrity Number ≥ 7 were used for gene expression analysis. RNA concentration in the samples was determined by NanoDrop^™^ 2000 spectrophotometer (Thermo Fisher Scientific). The nCounter Mouse Glial Profiling Panel (NanoString Technologies, Inc., Seattle, WA) was used to assess the expression of 770 glia specific genes in samples containing 100 ng of total RNA. This analysis was carried out by the Genome Technology Center at NYU Grossman School of Medicine using nCounter MAX Analysis System. Gene expression data were analyzed using nSolver Analysis Software v4.0 (NanoString Technologies Inc.) and included only genes consistently producing a read of ≥ 25 counts per brain. Raw counts were normalized using 13 internal reference genes as described before [77]. Gene expression heatmaps were created using the nSolver Analysis Software v4.0, which also was used for cluster analysis of individual animals. In addition, we computed a fold change for each analyzed gene in 22L-infected animals relative to their *APOE*-matched NBH inoculated controls.

### Reverse Transcription Quantitative Polymerase Chain Reaction (RT-qPCR) analysis

Two micrograms of total RNA per brain were reverse transcribed into cDNA using an iScript^™^ Advanced cDNA Synthesis Kit (Bio-Rad Laboratories, Hercules, CA). Sequences of primers used to determine the expression of target and housekeeping genes are listed in Table 3. Their amplification efficiency was vetted and optimized to remain within the 90% to 110% range. The qPCR was performed using SsoAdvanced Universal SYBR Green Supermix on the CFX96 Real-Time System (Bio-Rad Laboratories). Differences in gene expression were analyzed using ΔΔCt method [65, 77].

### Statistical analysis

Disease latency time was analyzed using Kaplan-Meier estimator and the differences across *APOE* genotypes were compared using Log-Rank test. Differences in the total scrapie score and its components were tracked longitudinally and analyzed by repeated measures analysis of variance (ANOVA). Data distribution of individual, quantitative metrics was vetted using Kolmogorov-Smirnov and Shapiro-Wilk tests to assess conformity with the normal distribution pattern. Differences across multiple data sets were first analyzed with one-way ANOVA, which was followed by Holm-Sidak’s post hoc test, comparing pairs of individual experimental groups. Sex differences within individual experimental groups were tested with the help of unpaired t-test with Welch’s correction. Differences in the PrP signal distribution across 14 fractions resulting from sucrose density gradient centrifugation of brain homogenate were determined using the Kolmogorov-Smirnov test between each pair of experimental groups. GraphPad Prism (v10.4.1 for Windows, GraphPad Software, Boston, MA) was used for all statistical analyses and graph making.

## Results

### APOE genotype modulates latency, symptom progression, and pathology burden in a mouse model of prion disease

**Figure 1:**
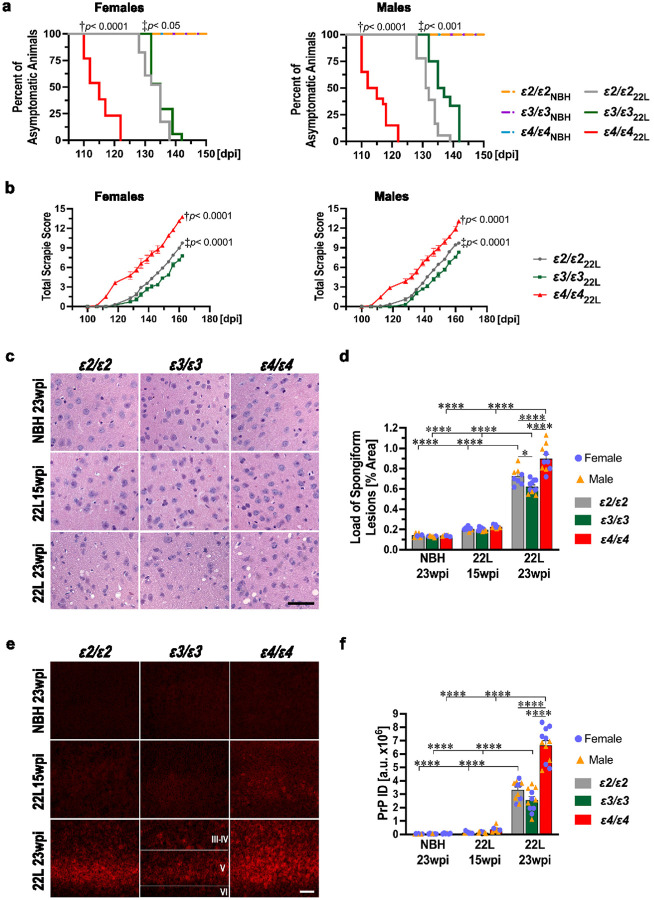
The *APOE* genotype differentially affects prion disease latency time, symptoms severity, and pathology burden. **(a)** Plots of Kaplan-Meier estimates of the prion disease latency time and **(b)** those of the total scrapie score for animals of indicated sex, the *APOE* genotype, and inoculum (22L or NBH). X-axes denote number of days post inoculation (dpi). **(c)** Micrographs of the coronal sections through the M1 motor cortex (bregma +1.4mm), which were stained with hematoxylin/eosin and demonstrate severity of spongiform lesion and **(e)** those through the S1 somatosensory cortex (bregma 0.0mm), which were immunostained against the prion protein. **(d)** Unbiased quantification of the spongiform lesion load in the M1 cortex and **(f)** that of integrated density (ID) of anti-PrP immunostaining in the S1 cortex. Animals are grouped by the *APOE* genotype, inoculum (NBH or 22L), and survival time expressed as the number of weeks post inoculation (wpi). **(a)** Kaplan-Meier estimates n=8–14 or n=12–23 animals per *APOE* genotype for NBH and 22L inoculated groups, respectively. †*p*< 0.0001 denotes significance between *ε4/ε4*_22L_ and *ε3/ε3*_22L_ or *ε2/ε2*_22L_, while ‡*p*< 0.05 or ‡*p*< 0.001 between *ε3/ε3*_22L_ and *ε2/ε2*_22L_ (Log-Rank test). Differences between 22L and NBH groups are not shown, but they are significant at *p*< 0.0001 for any pairwise comparison. **(b)** Values represent mean ± SEM from 4–12 animals per sex and *APOE* genotype. †*p*< 0.0001 denotes significance between *ε4/ε4*_22L_ and *ε3/ε3*_22L_ or *ε2/ε2*_22L_, while ‡*p*< 0.0001 between *ε2/ε2*_22L_ and *ε3/ε3*_22L_ (repeated measures ANOVA). **(d, f)** Values represent mean + SEM from n=5–9 and n=8–12 animals per *APOE* genotype in NBH_23wpi_ and 22L_15wpi_ groups and 22L_23wpi_ groups, respectively. *p*< 0.0001 (one-way ANOVA); *p< 0.05, and ****p< 0.0001 (Holm’s-Sidak’s post hoc). Non-significant differences not shown. **(e)** Roman numerals denote neuronal layers of the isocortex. Scale bars: 50mm in **(c)** and 100mm in **(e)**.

The latency period of prion disease was determined through serial locomotor testing, and the differences across animals of different *APOE* genotypes were compared using the Kaplan-Meier estimator. The *ε4/ε4*_22L_ mice were the first to show neurological signs of the prion disease with a median latency of 115.0 days in females and 113.5 days in males. The *ε2/ε2*_22L_ mice were second to be affected with a median latency of 135.0 days in females and 131.5 days in males, followed by *ε3/ε3*_22L_ mice, where the median latency was 136.0 days for both sexes. Differences across *APOE* genotypes both for female and male animals were statistically significant (Fig. 1a), while female and male mice of the same *APOE* genotype showed no significant differences (Supplementary File 1; Fig. S1). To quantify progression and severity of neurological symptoms we conducted serial assessments using the Total Scrapie Score (TSS) in a subset of 22L-infected mice. The TSS is a 15-point cumulative scale, accounting for five scorable behavioral metrics: animal alertness (somnolence), hind limb weakness, posture (kyphosis), walking competency, and body condition. Both *ε4/ε4*_22L_ females and males showed the most aggressive tempo of disease progression compared to mice of other *APOE* genotypes. At 23 weeks post inoculation (wpi.), which was the final time point of the experiment, *ε4/ε4*_22L_ females scored on average 13.8 ± 0.1 pts. on the TSS scale (*p* < 0.0001 vs. *ε2/ε2*_22L_ and *ε3/ε3*_22L_), while *ε4/ε4*_22L_ males scored 13.1 ± 0.1 pts. (*p* < 0.0001 vs. *ε2/ε2*_22L_ and *ε3/ε3*_22L_) (Fig. 1b). *ε2/ε2*_22L_ mice scored worse than *ε3/ε3*_22L_ mice, with females and males scoring an average of 9.8 ± 0.1 pts. (*p* < 0.0001 vs. *ε3/ε3*_22L_) and 9.7 ± 0.2 pts. (*p* < 0.0001 *ε3/ε3*_22L_), respectively. At 23 wpi. TSS scores in *ε3/ε3*_22L_ females and males were 7.8 ± 0.1 pts. and 8.3 ± 0.1 pts., respectively. The *ε4* > > *ε2* > *ε3 APOE* allele gradient effect was consistent across all five individual components of the TSS, with the most prominent differences in respect to alertness, walk, and body condition (Supplementary File 1: Fig. S2 a, b). Differences in the TSS and its individual components between *APOE* genotype matched female and male mice were not statistically significant.

To ensure that differences in disease latency and the tempo of symptoms progression across mice of different *APOE* genotypes are not caused by variable accumulation of PrP^Sc^ in the LRS, we assayed the level of PK-resistant PrP^Sc^ in the spleen homogenate. In presymptomatic 22L-infected mice, which were killed at 15 wpi., PrP^Sc^ was readily detectable in the spleen, but its level did not significantly differ across *APOE* genotypes (Supplementary File 1: Fig. S3 a, b). In NBH-inoculated control animals no PK-resistant PrP^Sc^ signal was detectable.

To determine the effect of the *APOE* genotype on the burden of prion pathology in the brain we quantified the load of spongiform lesions in the M1 motor cortex. Presymptomatic 22L-infected mice showed only a limited number of spongiform lesions. In contrast, symptomatic mice, euthanized at 23 wpi. featured numerous spongiform lesions, which load was significantly affected by the *APOE* genotype. In *ε4/ε4*_22L_ mice the spongiform lesion load was 1.24- and 1.44-fold higher than that in *ε2/ε2*_22L_ and *ε3/ε3*_22L_ animals (*p* < 0.0001), respectively; with the difference between the two latter groups being significant (*p* < 0.05) (Fig. 1c, d). We also analyzed, the integrated density (ID) of anti-PrP immunostaining in the S1 somatosensory cortex. Not presymptomatic, but symptomatic 22L-infected mice showed a significant increase in the anti-PrP ID values relative to NBH controls, and this effect was *APOE* genotype dependent (Fig. 1e, f). *ε4/ε4*_22L_ mice had 2.0 and 2.6- fold higher values of anti-PrP ID compared to *ε2/ε2*_22L_ and *ε3/ε3*_22L_ animals (*p* < 0.0001), respectively; with the difference between the latter two groups not reaching statistical significance. Differences in the spongiform lesion load and the anti-PrP ID values between female and male animals for matching *APOE* genotypes, inoculum type, and the survival time were not statistically significant (Supplementary File 1: Fig. S4 a, b).

### The APOE ε4 allele is associated with greater PrP accumulation, PrP^Sc^ conversion, and aggregation

**Figure 2:**
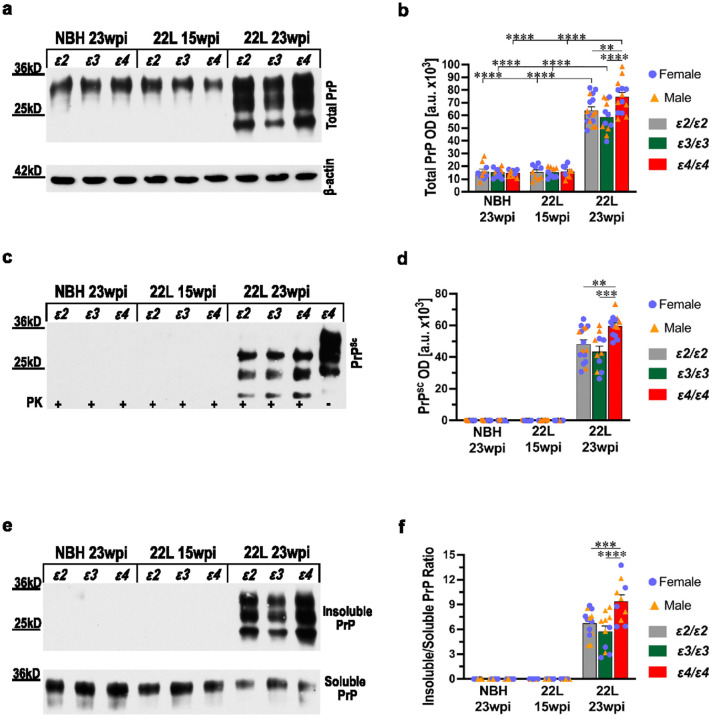
The *APOE ε4* allele is associated with increased PrP^Sc^ accumulation. Shown are immunoblot analyses for **(a)** the total PrP protein, **(c)** proteinase K (PK) resistant PrP^Sc^ conformer, and **(e)** total PrP separated into detergent insoluble and soluble fractions in the brain cortex homogenate from mice of indicated *APOE* genotypes, which were inoculated with normal brain homogenate (NBH) or 22L scrapie strain and killed 15 or 23 weeks post inoculation (wpi). Also included are an immunoblot for β-actin in **(a)** and a PK undigested sample of brain cortex homogenate in **(c)**, which were used as controls for equal protein load and protein-band weigh-shift resulting from PK digestion, respectively. Shown are results of densitometric quantification of the protein band optical densities (OD) for **(b)** the total PrP protein, **(d)** the PrP^Sc^ conformer, and **(f)** the insoluble / soluble PrP ratio. These analyses show a significant increase in the total PrP level, the appearance of PrP^Sc^ and detergent insoluble PrP in 22L_23wpi_ groups compared to NBH_23wpi_ and 22L_15wpi_ animals. The greatest increase in all three metrics is seen in *ε4/ε4*_22L_ mice at 23 wpi. **(b, d, f)** Shown are mean values + SEM from 6 to 10 mice per *APOE* genotype in NBH_23wpi_ and 22L_15wpi_ groups and from 10 to 16 mice per *APOE* genotype in 22L_23wpi_ groups along with data points representing single female and male animals. *p*< 0.0001 (one-way ANOVA); **p< 0.01, ***p< 0.001, and ****p< 0.0001 for post-hoc comparison (Holm’s-Sidak’s test). Non-significant differences are not shown on the graphs.

Prion pathology is invariably associated with an increase in the brain total PrP protein level and the appearance of its PK-resistant conformer PrP^Sc^. The total PrP level showed no differences across NBH-inoculated control mice of various *APOE* genotypes and no significant increase in presymptomatic 22L-infected mice, at 15 wpi. In contrast, symptomatic 22L-infected mice, euthanized at 23 wpi., featured a marked increase in the total brain PrP level by ~ 4- to ~ 5- folds compared to *APOE* matched NBH controls (*p* < 0.0001) (Fig. 2a, b). The highest total PrP level was found in *ε4/ε4*_22L_ mice, and it was 1.2- and 1.3-fold higher than those in *ε2/ε2*_22L_ (*p* < 0.01) and *ε3/ε3*_22L_ (*p* < 0.0001) animals, respectively; with the difference between the two latter groups not reaching statistical significance. The PrP^Sc^ conformer was undetectable by the PK-digestion assay in the brains of NBH-controls and presymptomatic 22L-infected mice. In contrast, in symptomatic 22L-infected mice PrP^Sc^ was abundantly detected, and its level was significantly potentiated by the presence of the *ε4* allele (Fig. 2c, d). *ε4/ε4*_22L_ mice featured 1.24- and 1.37-fold higher PrP^Sc^ level compared to *ε2/ε2*_22L_ (*p* < 0.01) and *ε3/ε3*_22L_ mice (*p* < 0.001), respectively; with the difference between the two latter groups not reaching statistical significance.

Using the detergent solubility assay, we characterized solubility changes the PrP protein undergoes during the PrP^C^ to PrP^Sc^ transformation. Brains from NBH and presymptomatic 22L-infected mice showed no evidence of detergent insoluble PrP protein. In symptomatic, 22L-infected mice detergent insoluble PrP, was not only abundantly present, but its amount well exceeded that detected in the detergent soluble fraction. The highest ratio of detergent insoluble to detergent soluble PrP was in *ε4/ε4*_22L_ mice (9.4 ± 0.8) and it was significantly higher than those in *ε2/ε2*_22L_ mice (6.7 ± 0.4) (*p* < 0.001) and in *ε3/ε3*_22L_ mice (5.7 ± 0.7) (*p* < 0.0001) (Fig. 2e, f). The difference between *ε2/ε2*_22L_ and *ε3/ε3*_22L_ mice was not statistically significant. Differences in the total PrP level, PrP^Sc^ level, and the insoluble to soluble PrP ratio between female and male mice for matching *APOE* genotype, inoculum, and survival time were not statistically significant (Supplementary File 1: Fig. S5 a-c).

**Figure 3:**
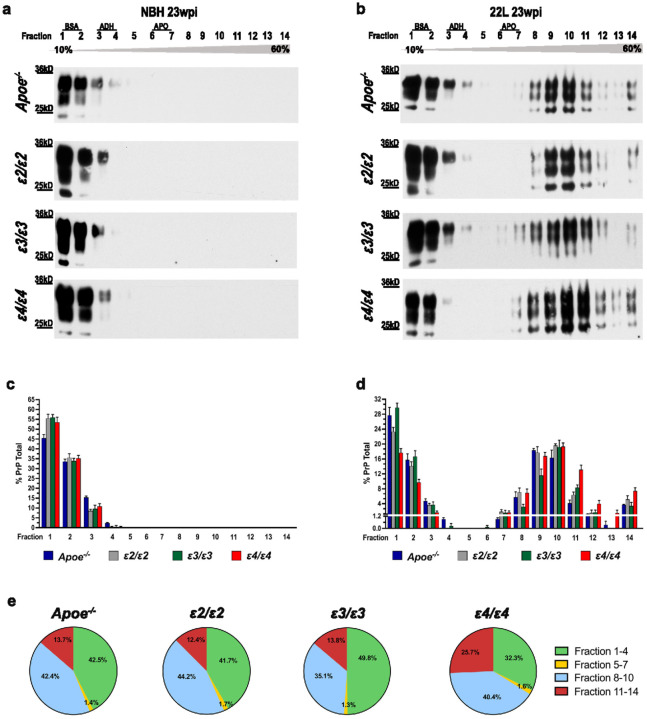
The *APOE ε4* allele is associated with increased PrP oligomerization. Shown is immunoblot analysis of the PrP protein in the brain homogenate, which was separated into 14 fractions by velocity sedimentation using a 10%−60% sucrose gradient. Mice of indicated *APOE* genotypes were inoculated with **(a)** normal brain homogenate (NBH) or **(b)** 22L scrapie strain and killed 23 weeks post inoculation (wpi). Molecular weight markers: bovine serum albumin (BSA) (68 kDa), alcohol dehydrogenase (ADH) (150 kDa) and apoferritin (APO) (443 kDa) were detected within indicated fractions. Shown are the results of densitometric analysis of the PrP protein band optical densities (OD) detected across the 14 fractions in NBH **(c)** or 22L inoculated animals **(d)**. Values represent a percentage of the prion protein band OD in each fraction relative to the sum of OD in 1–14 fractions. Shown is mean + SEM from 3–8 animals per *APOE* genotype and inoculum. In NBH inoculated animals PrP is detectable only in fractions 1–4, while in 22L infected mice also in fractions 8–10 and 11–14, what indicates formation of PrP oligomeric complexes during prion infection. This effect is *APOE* genotype dependent with *ε4/ε4*_22L_ mice showing the highest level of PrP oligomers in fractions 11–14. Two-sample Kolmogorov-Smirnov test was used for pairwise analysis of differences in the distribution of the PrP signal across the 14 fractions: *p*< 0.0001 for *ε4/ε4*_22L_ vs. *ε3/ε3*_22L_, *ε2/ε2*_22L_ or *Apoe*^−/−^_22L_ and *p*< 0.01 for *ε3/ε3*_22L_ vs. *ε2/ε2*_22L_. Differences between *ε3/ε3*_22L_ or *ε2/ε2*_22L_ vs. *Apoe*^−/−^_22L_ were not significant. See Supplementary File 2, Table S1. **(e)** Pie chart visualization of the *APOE* genotype effect on the PrP oligomerization in 22L infected mice. PrP OD signals in fractions 1–4, 5–7, 8–10, and 11–14 were summed, averaged across individual animals, and expressed as fractions of the total.

We also investigated the effect of *APOE* genotype on PrP oligomerization by subjecting brain cortex homogenate to sucrose gradient centrifugation. The resulting 14 fractions were individually collected and resolved using SDS-PAGE under reducing conditions and immunoblotted for PrP (Fig. 3a, b). Brains from NBH-inoculated and symptomatic, 22L-infected *ε2/ε2*, *ε3/ε3*, and *ε4/ε4* mice were examined along with those from *Apoe*^*−/−*^ mice. In NBH controls, the PrP signal was detectable only in fractions 1–4 and its distribution showed no statistically significant differences across *APOE* genotype (Fig. 3c; Supplementary File 2: Table S1). In contrast, in 22L-infected mice, the PrP signal was detected across all 14 fractions, and its distribution bore a significant *ε4* effect (Fig. 3d). The *ε4/ε4*_22L_ mice featured the most pronounced, right-sided shift in the PrP signal distribution across the 14 fractions compared to any other 22L-infected *APOE* genotype or *Apoe*^*−/−*^ animals (*p* < 0.0001). Differences in the PrP signal distribution pattern between *Apoe*^*−/−*^_22L_ and *ε2/ε2*_22L_ or *ε3/ε3*_22L_ mice were insignificant. A statistically significant difference was noted only between *ε2/ε2*_22L_ and *ε3/ε3*_22L_ mice (*p* < 0.01) owing to the most left-sided shift in the signal distribution in the latter group. To better visualize the *ε4* effect in 22L-infected mice we grouped the fractions into four clusters 1–4, 5–7, 8–10, and 11–14, and showed the proportional contribution of each cluster to the total PrP signal in all 14 fractions using pie charts (Fig. 3e). In *ε4/ε4*_22L_ mice, the cluster 11–14 contributed 25.7% of the total PrP signal, while for comparison in *ε2/ε2*_22L_, *ε3/ε3*_22L_, and *Apoe*^*−/−*^_22L_ groups its contribution ranged from 12.4% to 13.8%. Conversely, the cluster 1–4 in *ε4/ε4*_22L_ mice constituted 32.3% of the total PrP signal, while in *ε2/ε2*_22L_, *ε3/ε3*_22L_, and *Apoe*^*−/−*^_22L_ groups its contribution ranged from 41.7% to 49.8%. This experiment demonstrates that while PrP oligomerization is an inherent feature of the prion proteinopathy, it is promoted only in the presence of the *ε4* allele, as there are no significant differences between *Apoe*^*−/−*^_22L_ and *ε2/ε2*_22L_ or *ε3/ε3*_22L_ animals.

### Increase in the apoE protein level and formation of the PrP/apoE complexes during prion infection is APOE genotype dependent

**Figure 4:**
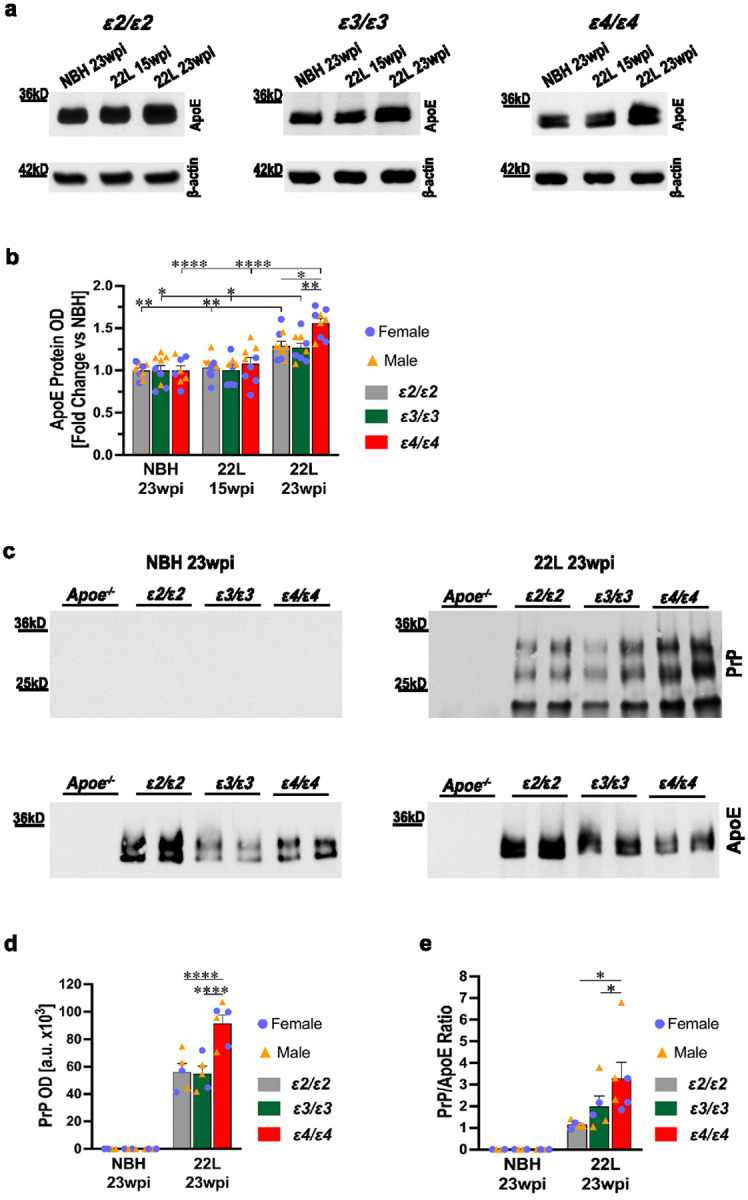
ApoE protein accumulates during prion infection and forms complexes with PrP in the *APOE* allele dependent manner. **(a)** Shown are immunoblot analyses of the apoE protein level in the brain cortex in animals of indicated *APOE* genotype, inoculum type, and survival time. Also included are immunoblots for b-actin used as a loading control in all experiments. **(b)** Densitometric quantification of apoE protein band optical densities (OD) in 22L infected mice at 15 and 23 weeks post inoculation (wpi) expressed relative to NBH-inoculated controls for matching *APOE* genotype. Significant increase in the apoE level is seen in 22L infected mice at 23 wpi but not at 15 wpi and is the highest in *ε4/ε4*_22L_ animals. **(c)** Shown are immunoblot analyses of PrP/apoE complexes in mice of indicated *APOE* genotype, inoculum type, and survival time. The complexes were immunoprecipitated using the HJ15.3 anti-apoE mAb from the brain cortex homogenate, resolved by SDS-PAGE under reducing conditions, and dissociated PrP was detected using 6D11 anti-CD230 clone. Included are immunoblots for apoE on membranes, which were stripped and re-probed using goat polyclonal anti-human apoE antibody. Shown are the results of densitometric quantification of **(d)** PrP protein band optical densities (OD) and **(e)** the ratio of PrP to apoE OD in PrP/apoE complexes immunoprecipitated with HJ15.3 anti-apoE mAb. Values represent mean + SEM from 8 to10 mice per group in **(b)** and from 4 to 6 mice per group in **(d)** and **(e)** along with data points for single female and male animals. **(b)**, **(d)**, and **(e)** *p*< 0.0001 (one-way ANOVA); *p< 0.05, **p< 0.01, and ****p< 0.0001 for post-hoc comparison (Holm’s-Sidak’s test). Non-significant differences are not shown on the graphs.

Consistently with previously published data, we found a significant effect of the *APOE* genotype on the brain apoE protein level in NBH control mice. *ε2/ε2*_NBH_ animals featured 1.2- and 1.5- fold higher apoE level compared to *ε3/ε3*_NBH_ (*p* < 0.05) and *ε4/ε4*_NBH_ mice (*p* < 0.0001) (Fig. 4a; Supplementary File 1: Fig. S6), respectively; with the difference between *ε3/ε3*_NBH_ and *ε4/ε4*_NBH_ mice being statistically significant (*p* < 0.01). Prion infection gave rise to a significant increase in the brain apoE level in symptomatic (23 wpi.) but not in presymptomatic (15 wpi.) animals (Fig. 4a, b). The magnitude of this increase varied across *APOE* genotypes, and it was the highest in *ε4/ε4*_22L_ mice, where the level of apoE protein rose 1.6-fold relative to *ε4/ε4*_NBH_ controls (*p* < 0.0001). Both in *ε2/ε2*_22L_ and *ε3/ε*3_22L_ mice the increase in apoE level was 1.3-fold relative to *ε2/ε2*_NBH_ (*p* < 0.01) and *ε3/ε3*_NBH_ (*p* < 0.05) controls, respectively. Animal sex had no significant effect on the brain apoE level, neither in NBH-controls nor in 22L-infected mice (Supplementary File 1: Fig. S7).

To determine whether apoE directly interacts with PrP, we immunoprecipitated the apoE/PrP complexes from the brain cortex homogenate using magnetic beads coated with HJ15.3 mAb, which reacts with the human apoE sequence. Captured complexes were resolved on SDS-PAGE under reducing conditions and the resulting monomeric PrP was detected using anti-CD230 clone 6D11 mAb (Fig. 4c). The PrP signal was detected in symptomatic *ε2/ε2*_22L_, *ε3/ε3*_22L_, and *ε4/ε4*_22L_ mice, but not in NBH-inoculated controls.

Optical density (OD) of the PrP protein band released from the complexes in *ε4/ε4*_22L_ mice was ~ 1.6-fold higher compared to *ε2/ε2*_22L_ or *ε3/ε3*_22L_ mice (*p* < 0.0001) (Fig. 4d), while the difference between the latter two groups was not statistically significant. We also quantified the PrP/apoE OD ratio by dividing the PrP protein band OD by that of apoE, which was detected on the same membrane as PrP, following membrane stripping and re-probing with goat polyclonal anti-human apoE antibody. The PrP/apoE OD ratio in *ε4/ε4*_22L_ mice was 2.8- and 1.7- fold higher than those in *ε2/ε2*_22L_ and *ε3/ε3*_22L_ mice (*p* < 0.05) (Fig. 4e), respectively, while the difference between the latter two groups was not statistically significant. As additional negative experimental controls, we used brain cortex homogenate from *Apoe*^*−/−*^_NBH_ and *Apoe*^*−/−*^_22L_ animals, in which no apoE/PrP complexes were detected. Our findings indicate that the apoE protein directly interacts with PrP but only in prion disease and not under physiological conditions.

### Microglia activation is differentially regulated by the APOE genotype

**Figure 5:**
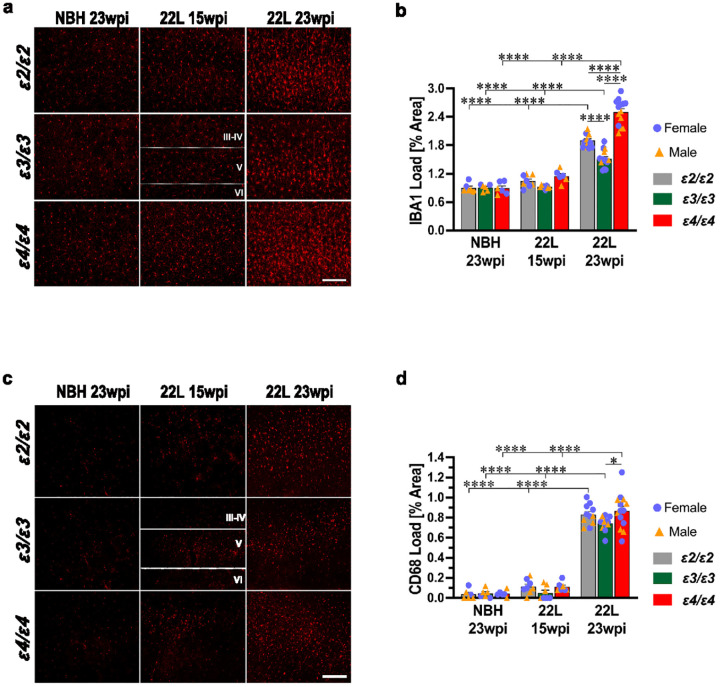
*APOE* genotype differentially affects microglia activation during prion infection. Shown are representative micrographs of the coronal sections through the S1 somatosensory cortex (bregma 0.0 mm), which were immunostained against microglia specific markers: **(a)** ionized calcium adaptor protein 1 (IBA1) and **(c)** cluster of differentiation 68 (CD68) in mice of indicated *APOE* genotype, inoculum type, and survival time. Unbiased quantification of **(b)** IBA1 and **(d)** CD68 positive microglia load in the S1 somatosensory cortex. Values represent mean + SEM from 6 to 8 mice per *APOE* genotype in NBH_23wpi_ and 22L_15wpi_ groups and from 11 to 14 mice per *APOE* genotype in 22L_*23wpi*_ groups. Data points represent single female and male animals. **(b)**, **(d)** *p*< 0.0001 (one-way ANOVA); *p< 0.05, and ****p< 0.0001 for post-hoc comparison (Holm’s-Sidak’s test). Non-significant differences are not indicated on the graphs. Roman numerals in **(a)** and **(c)** denote neuronal layers of the brain isocortex. Scale bars: 150mm in **(a)** and **(c)**.

Microglia activation was characterized by unbiased quantification of IBA1- and CD68-positive microglia load in the S1 somatosensory cortex alongside transcriptomic analysis of microglia specific genes. Presymptomatic, 22L-infected mice (15 wpi.) already showed a modest, but statistically insignificant increase in the IBA1 and CD68 load relative to NBH-inoculated controls. A robust and significant increase in the IBA1 and CD68 load was observed in symptomatic 22L-infected mice (23 wpi.), and this effect was *APOE* genotype dependent (Fig. 5a–d). The strongest activation of microglia was noted in *ε4/ε4*_22L_ mice, which had a 1.32- and 1.64-fold greater IBA1 load relative to the *ε2/ε2*_22L_ (*p* < 0.0001) and *ε3/ε3*_22L_ (*p* < 0.0001) mice, respectively. Likewise, *ε4/ε4*_22L_ mice showed 1.04-fold and 1.17-fold greater CD68 load relative to *ε2/ε2*_22L_ (non-significant) and *ε3/ε3*_22L_ (*p* < 0.05) mice, respectively. The value of IBA1 load in *ε2/ε2*_22L_ mice was significantly higher than that in *ε3/ε3*_22L_ mice (*p* < 0.0001), while the difference in the CD68 load insignificantly favored *ε2/ε2*_22L_ animals. It is noteworthy that the increase in the IBA1 load in symptomatic 22L-infected mice relative to their *APOE*-matched NBH controls ranged between 1.7-fold and 2.8-fold, while the increase in the CD68 load ranged between 16.4-fold and 22.7-fold. Differences in the IBA1 and CD68 load values between female and male animals for matching *APOE* genotypes, inoculum type, and the survival time were not statistically significant (Supplementary File 1: Fig. S8 a, b).

**Figure 6:**
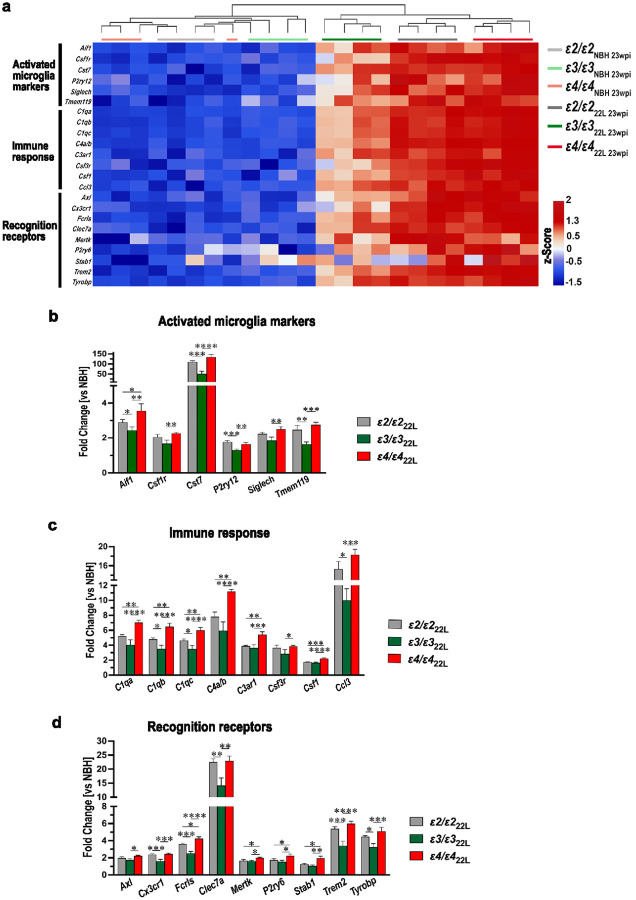
Microglia transcriptomic response during prion infection is differentially regulated by the *APOE* genotype. **(a)** Shown is the transcript heatmap of nanoStringTM nCounter^®^ expression data from animals of indicated *APOE* genotype and inoculum type at 23 weeks post inoculation (wpi). Microglia specific genes are vertically arranged into three groups: activated microglia markers, immune response, and recognition receptors. Results of hierarchical cluster analysis for all genes are displayed above the heatmap. Shown is fold change of nanoStringTM nCounter^®^ values in 22L_23wpi_ animals relative to their *APOE*-matched NBH_23wpi_ controls for **(b)** activated microglia gene markers, **(c)** microglia immune response genes, and **(d)** recognition receptors genes. Values represent mean + SEM from four animals per *APOE* genotype. **(b)**, **(c)**, and **(d)** *p*< 0.0001 for all analyzed genes except for *Stab1 p*< 0.001 (one-way ANOVA); *p< 0.05, **p< 0.01, ***p< 0.001, and ****p< 0.0001 for post-hoc comparison (Holm’s-Sidak’s test). Non-significant differences are not shown on the graphs.

Transcriptomic analysis included microglial genes, which were significantly upregulated in at least one *APOE* genotype within the symptomatic 22L-infected group compared to *APOE*-matched NBH controls (Supplementary File 2: Table S2). Significantly upregulated genes were grouped into three functional categories 1) activated microglia markers (*Aif1, Csf1r, Cst7, P2ry12, Siglech*, and *Tmem119*), 2) genes involved in immune response (*C1qa, C1qb, C1qc, C4a/b, C3ar1, Csf3r, Csf1*, and *Ccl3*), 3) and those encoding various microglia recognition receptors (*Axl, Cx3cr1, Fcrls, Clec7a, Mertk, P2ry6, Stab1*, *Trem2*, and *Tyrobp*). Hierarchical cluster analysis of all genes showed no systematic clustering across individual NBH animals. In contrast, 22L-infected animals featured a strong hierarchical signal (Fig. 6a). First, the animals clustered within their *APOE* genotypes, and then *ε2/ε2*_22L_ and *ε4/ε4*_22L_ animals clustered together separate from *ε3/ε3*_22L_ animals. We also compared differences in the fold increase of individual gene expression across *APOE* genotypes. *ε4/ε4*_22L_ mice showed significantly higher upregulations of all genes compared to *ε3/ε3*_22L_ mice and *Aif1, C1qa, C1qb, C1qc, C4a/b, C3ar1, Csf1, Fcrls, Mertk, P2ry6, Stab1* genes compared to *ε2/ε2*_22L_ mice (Fig. 6b–d). *Aif1, Cst7, P2ry12, Tmem119, C1qb, C1qc, Ccl3, Cx3cr1, Fcrls, Clec7a, Trem2*, and *Tyrobp* genes were expressed at significantly higher level in *ε2/ε2*_22L_ mice compared to *ε3/ε3*_22L_ mice. *Cst7, C4a/b, Ccl3*, and *Clec7a* were found to be upregulated at particularly high level (≥ 10-fold relative to NBH controls) in at least one of the *APOE* genotypes (Supplementary File 2: Table S2).

### APOE genotype differentially modulates activation of astrocytes during prion infection

**Figure 7:**
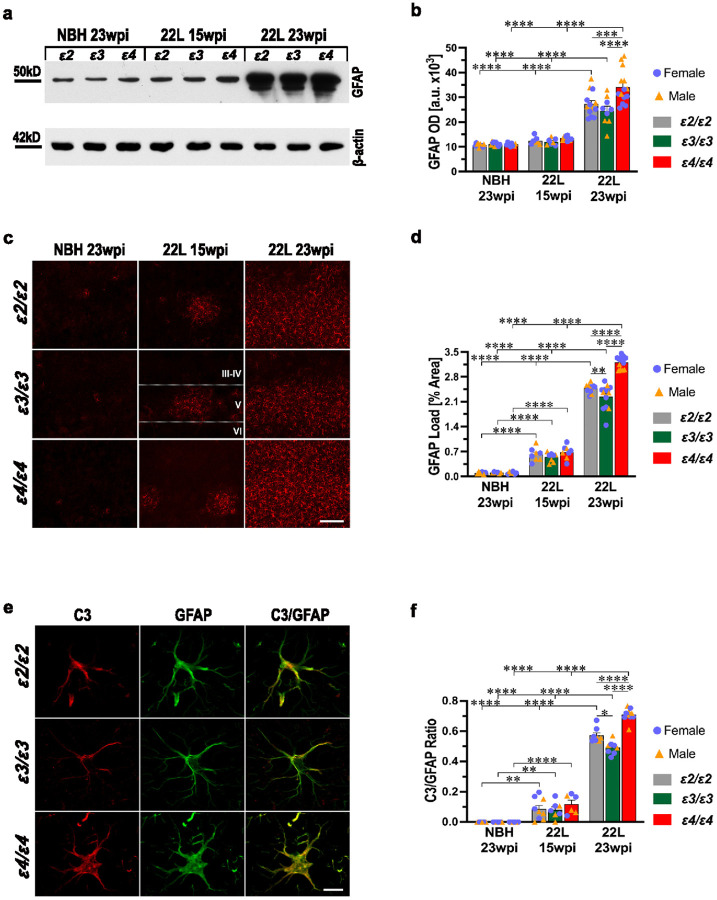
Prion-related astrogliosis is differentially affected by the *APOE* genotype. Shown are **(a)** immunoblot analysis of the glial fibrillary acidic protein (GFAP) in the brain cortex in animals of indicated *APOE* genotype, inoculum type, and survival time along with immunoblots for b-actin used as a loading control and **(b)** densitometric quantification of GFAP band optical densities (OD) exemplified in **(a)**. Shown are **(c)** representative microphotographs of the coronal sections through the S1 somatosensory cortex (bregma 0.0 mm), which were immunostained against GFAP and **(d)** unbiased quantification of GFAP positive astrocyte load in the S1 somatosensory cortex. Roman numerals in **(c)** denote cellular layers of the somatosensory cortex. **(e)** Shown are representative high magnification microphotographs of astrocytes double immunostained for the C3 complement protein and GFAP and **(f)** quantitative analysis of C3/GFAP ratio in the S1 cortex. Values in **(b)**, **(d)**, and **(f)** represent mean + SEM from 6 to 10 mice per group except for 22L_23wpi_ groups where the number of animals ranges from 8 to 14. Values for individual female and male animals are shown overlying the group bars. **(b)**, **(d)**, and **(f)** *p*< 0.0001 (one-way ANOVA); *p< 0.05, **p< 0.01, ***p< 0.001 and ****p< 0.0001 for post-hoc comparison (Holm’s-Sidak’s test). Non-significant differences are not indicated. Scale bars: 150mm in **(c)** and 10mm in **(e)**.

Astrocytic activation was characterized by determining changes in the GFAP protein level by quantitative immunoblotting, unbiased quantification of GFAP and C3-positive astrocyte load in the S1 somatosensory cortex and transcriptomic analysis of astrocyte specific genes. GFAP protein level showed no differences across *APOE* genotypes in NBH-inoculated controls. In presymptomatic 22L-infected mice (15 wpi.), it was modestly, albeit insignificantly increased (1.1–1.2-fold), while in symptomatic 22L-infected mice (23 wpi.) its level ranged between 2.2-fold and 3.1-fold relative to *APOE*-matched NBH controls (*p* < 0.0001) (Fig. 7a, b). Differences across *APOE* genotypes in symptomatic 22L-infected mice were statistically significant with *ε4/ε4*_22L_ mice featuring 1.3- and 1.4-fold higher GFAP protein level compared to *ε2/ε2*_22L_ (*p* < 0.001) and *ε3/ε3*_22L_ mice (*p* < 0.0001), respectively; while the difference between the latter two groups was not significant.

The load of GFAP positive astrocytes in the S1 somatosensory cortex was already significantly increased in presymptomatic 22L-infected mice (15 wpi.) (*p* < 0.0001) (Fig. 7c, d), but without any significant *APOE* genotype effect. Symptomatic 22L-infected mice (23 wpi.) featured further increase in the GFAP load, which ranged between 20.1- and 32.1-fold relative to NBH controls (*p* < 0.0001). *ε4/ε4*_22L_ mice had a 1.3- and 1.4-fold higher GFAP load compared to *ε2/ε2*_22L_ (*p* < 0.0001) and *ε3/ε3*_22L_ mice (*p* < 0.0001), respectively, and the difference between the latter two groups was statistically significant (*p* < 0.01). We also quantified the load of C3-positive astrocytes and analyzed it in relation to the GFAP load (Fig. 7e, f). In NBH-inoculated control mice, C3-positive astrocytes were absent. For the first time, expression of C3 in astrocytes was noted in presymptomatic 22L-infected mice, where the C3/GFAP ratio ranged between 0.08 and 0.11 across *APOE* genotypes (*p* < 0.01 to *p* < 0.0001 vs. NBH). In symptomatic 22L-infected mice, the C3 expression increased further with C3/GFAP ratio reaching values of 0.49 to 0.71 across *APOE* genotypes (*p* < 0.0001 vs. NBH or 22L at 15 wpi). Differences in the C3/GFAP ratio showed a significant *APOE*-genotype effect in symptomatic but not in presymptomatic animals. Symptomatic *ε4/ε4*_22L_ mice featured 1.2- and 1.4-fold higher values of the C3/GFAP ratio compared to *ε2/ε2*_22L_ (*p* < 0.0001) and *ε3/ε3*_22L_ mice (*p* < 0.0001), respectively; with the difference between the latter two groups also being statistically significant (*p* < 0.05). There were no statistically significant differences in respect to the GFAP protein level, the GFAP-positive astrocyte load, and the C3/GFAP ratio between female and male animals for matching *APOE* genotype, inoculum type, and survival time (Supplementary File 1: Fig. S9 a-c).

**Figure 8:**
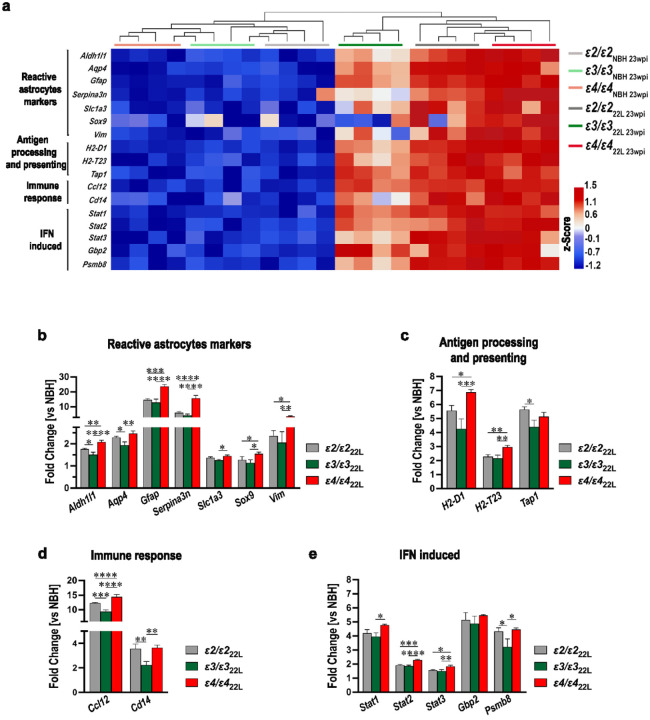
*APOE* genotype differentially regulates transcriptomic profile in astrocytes. **(a)** Shown is the transcript heatmap of nanoStringTM nCounter^®^ expression data from animals of indicated *APOE* genotype and inoculum type at 23 weeks post inoculation (wpi). Astrocyte specific genes are vertically arranged into four groups: markers of reactive astrocytes, antigen processing and presenting, immune response, and interferon (IFN) induced. Results of hierarchical cluster analysis for all genes are displayed above the heatmap. Shown is fold change of nanoStringTM nCounter^®^ values in 22L_23wpi_ animals relative to their *APOE*-matched NBH_23wpi_ controls for **(b)** reactive astrocytes gene markers, **(c)** antigen processing and presenting genes, **(d)** immune response genes and **(e)** IFN induced genes. Values represent mean + SEM from four animals per *APOE* genotype. **(b)**, **(c)**, **(d)** and **(e)** *p*< 0.0001 for all analyzed genes except for *Sox9 p*< 0.01; *p< 0.05, **p< 0.01, ***p< 0.001, and ****p< 0.0001 for post-hoc comparison (Holm’s-Sidak’s test). Non-significant differences are not shown on the graphs.

Transcriptomic analysis included those astrocytic genes, which were significantly upregulated in at least one *APOE* genotype in symptomatic 22L-infected mice relative to their *APOE*-matched NBH controls (Supplementary File 2; Tab S3). Analyzed genes were grouped into four functional categories: 1) markers of reactive astrocytes (*Aldh1l1, Aqp4, Gfap, Serpina3n, Slc1a3, Sox9, Vim*), 2) genes involved in antigen presenting and processing (*H2-D1, H2-T23, Tap1*), 3) genes involved in immune response (*Ccl12, Cd14*), and 4) those encoding astrocytic markers, whose expression is induced by interferons (*Stat1, Stat2, Stat3, Gbp2, Psmb8*). Hierarchical cluster analysis of all genes showed no systematic clustering across individual NBH-inoculated animals. *ε3/ε3*_22L_ mice clustered separately from *ε2/ε2*_22L_ and *ε4/ε4*_22L_ mice, which clustered together. All NBH animals clustered separately from 22L-infected animals (Fig. 8a). We also compared differences in the fold increase of individual genes across *APOE* genotypes. *ε4/ε4*_22L_ mice showed significantly greater expression of all genes compared to *ε3/ε3*_22L_ mice except for *Tap1* and *Gbp2* (Fig. 8b–e). *ε4/ε4*_22L_ mice also showed significantly greater expression of *Aldh1l1, Gfap, Serpina3n, Vim, H2-D1, H2-T23, Ccl12, Stat2*, and *Stat3* compared to *ε2/ε2*_22L_ mice. *Aldh1l1, Aqp4, Tap1, Ccl12, Cd14*, and *Psmb8* genes were upregulated at significantly higher level in *ε2/ε2*_22L_ mice compared to *ε3/ε3*_22L_ mice. *Gfap, Serpina3n, Vim*, and *Ccl12* were upregulated at particularly high level (≥ 10-fold relative to NBH controls) in at least one of the *APOE* genotypes (Supplementary File 2: Table S3).

### APOE genotype differentially regulates reciprocal proinflammatory crosstalk between microglia and astrocytes

**Figure 9:**
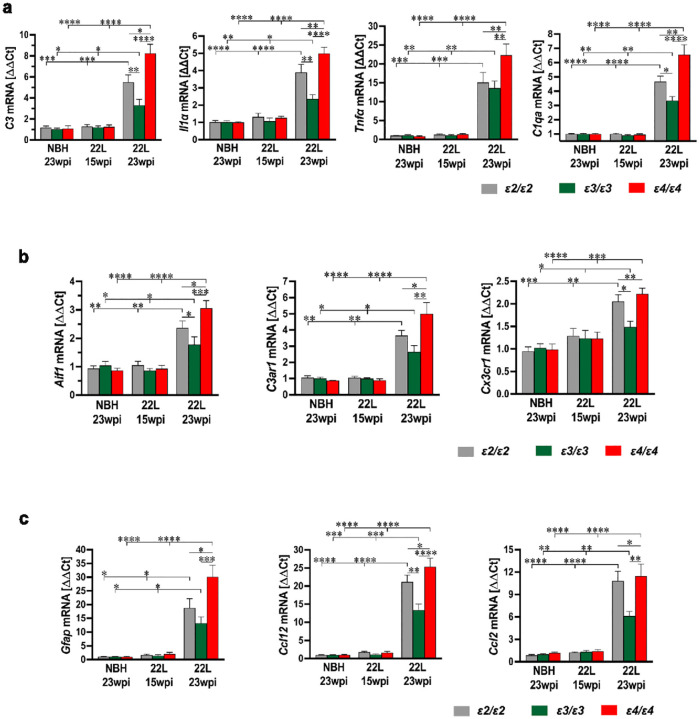
Reciprocal microglia-astrocyte activation is *APOE* genotype dependent. Shown are fold gene expression values for **(a)** genes underlying reciprocal microglia-astrocyte pro-inflammatory activation: *C3*, *Il1a*, *Tnfa* and *C1qa*, **(b)**neurodegenerative microglia markers *Aif1*, *C3ar1*, and *Cx3cr1*, and **(c)** reactive astrocyte markers *Gfap*, *Ccl12*, and *Ccl2*. Values represent mean + SEM of qRT-PCR readouts from 4 to 11 animals of indicated *APOE* genotype, inoculum, and survival time. **(a)**, **(b)**, and **(c)** *p*< 0.0001 (one-way ANOVA); *p< 0.05, **p< 0.01, ***p< 0.001, and ****p< 0.0001 for post-hoc comparison (Holm’s-Sidak’s test). Non-significant differences are not indicated.

Chronically reactive microglia secrete a triad of cytokines IL1-α, TNFα, and C1QA, which stimulate reactive astrocytes. These in turn secrete C3, which reciprocally stimulates neurodegenerative microglia. We explored the effect of *APOE* genotype on this pathway in prion infected mice using qRT-PCR (Fig. 9a–c). We compared the expression of *Il1α*, *Tnfα*, *C1qa*, and *C3* genes alongside expression of genes which are considered transcriptomic markers of neurodegenerative microglia phenotype (*Aif1*, *C3ar1*, and *Cx3cr1*) and those specifically associated with chronically reactive astrocytes (*Gfap*, *Ccl12*, and *Ccl2*). No changes in expression level of any of these genes were found in presymptomatic 22L-inoculated mice (15 wpi.) compared to NBH-inoculated controls for matching *APOE* genotypes. In contrast, symptomatic 22L-inoculated mice (23 wpi.) showed significant upregulation of all interrogated genes with significant differences across the *APOE* genotypes. The highest expression of all the genes was found in *ε4/ε4*_22L_ mice with differences between *ε4/ε4*_22L_ mice and *ε2/ε2*_22L_ and *ε3/ε3*_22L_ mice being statistically significant for all genes (*p* < 0.05 to *p* < 0.0001) except for *Cx3cr1* (*ε4/ε4*_22L_ vs. *ε3/ε3*_22L_). Expression of *Il1α*, *C1qa*, *C3, Aif1*, *Cx3cr1 and Ccl12* genes was significantly higher in *ε2/ε2*_22L_ mice compared to *ε3/ε3*_22L_ animals (*p* < 0.05 to *p* < 0.01).

## Discussion

By infecting *APOE*-TR mice with 22L mouse adapted scrapie strain, we identified a differential effect of human *APOE* alleles on prion induced neurodegeneration. *ε4/ε4*_22L_ mice featured the shortest disease latency, the fastest progression of neurological symptoms, the worst neurological score at the end of the study, and the highest load of spongiform lesions, PrP^Sc^ level, and neuroinflammatory response. In addition, we found that *ε2/ε2*_22L_ mice performed worse in respect to behavioral, neuropathological, and neuroinflammatory metrics compared to *ε3/ε3*_22L_ animals, which suggests the *ε2* allele might be a disadvantageous rather than protective determinant in prion pathology. Examination of spleens and brains of presymptomatic mice euthanized at 15 wpi., showed no evidence of differential effect of *APOE* alleles on PrP^Sc^ accumulation in the LRS or early brain pathology. This indicates prion neuroinvasion is independent of the *APOE* polymorphism and the variance we observed in disease outcomes results from differential effect of the *APOE* polymorphism on ensuing brain pathology. This is an important observation since apoE is expressed by the spleen’s dendritic cells [5]], which are known to replicate PrP^Sc^ and constitute its reservoirs outside the central nervous system [10, 83].

Conformational transformation of PrP^C^ into PrP^Sc^ is central to prion pathogenesis and involves several physicochemical changes within the PrP protein, which include reduced detergent solubility, oligomerization, acquisition of proteolytic resistance, and accumulation [30, 40, 41, 78, 81, 115]. *ε4/ε4*_22L_ mice featured significantly higher level of the total brain PrP, confirmed both by quantitative immunohistochemistry and Western immunoblotting, PK-resistant PrP^Sc^, and insoluble PrP fraction compared to *ε2/ε2*_22L_ and *ε3/ε3*_22L_ animals, in which values of these metrics were similar. Characterization of PrP oligomeric assemblies performed using sucrose density gradient centrifugation of brain homogenate detected PrP signal only in fractions 1–4 in NBH inoculated controls while in 22L infected mice the PrP signal was predominantly present in fractions 8–14. *Apoe*^*−/−*^_22L_, *ε2/ε2*_22L_, and *ε3/ε3*_22L_ mice showed similar pattern of PrP signal distribution across all 14 fractions, what suggests apoE is not a prerequisite for PrP oligomer formation since these are detectable in *Apoe*^*−/−*^_22L_ mice. However, *ε4/ε4*_22L_ animals featured a distinctly different PrP distribution pattern characterized by significantly increased signal in fractions 11–14, which represent higher order oligomers (+ 10-mers). This finding indicates that apoE4 isoform effectively promotes PrP oligomerization.

Consistent with several published studies, we found the brain level of apoE protein is *APOE* genotype dependent, with *ε2/ε2*_NBH_ and *ε4/ε4*_NBH_ animals representing opposite ends of the spectrum [28, 63, 109, 116]. The mechanism(s) underlying this phenomenon have not been fully elucidated, though differential receptor mediated clearance of various apoE isoforms has been postulated to play a central role. We previously showed that prion pathology is associated both with an increase in the brain apoE level and cell-type shift in apoE expression [77]. While under physiological conditions the bulk of brain apoE is produced by resting or A0 astrocytes, their activation is associated with reduced apoE expression [77]. Conversely, while resting (M0) microglia do not produce apoE, de-repression of apoE translation is an unique characteristic of their reactive states commonly referred to as disease-associated microglia (DAM) or microglia neurodegenerative phenotype (MGnD) [49, 53]. In all three *APOE*-TR lines, prion pathology was associated with increase in the total brain apoE level, but the magnitude of this effect was *APOE*-genotype dependent. While *ε2/ε2*_22L_, and *ε3/ε3*_22L_ mice featured a similar fold change relative to their *APOE* genotype matched NBH controls, the relative increase in *ε4/ε4*_22L_ mice was significantly higher. Notably *ε4/ε4*_22L_ animals featured the highest degree of microglia and astrocyte activation compared to *ε2/ε2*_22L_, and *ε3/ε3*_22L_ mice, what reasonably can explain the highest relative increase in apoE level in this line.

Using immunoprecipitation assay, we found apoE protein and disease-altered PrP form complexes, which become dissociated under reducing conditions. ApoE/PrP complexes were immunoprecipitated using HJ15.3 anti-apoE clone [44, 60, 103] and detected using 6D11 anti-PrP clone [93, 102]. Interestingly, the immunoprecipitation experiment did not work in reverse where 6D11 and HJ15.3 clones were used as the capture and the detection antibodies, respectively. This suggests, binding of apoE to PrP might hinder the 6D11 epitope comprised of residues 97–100 (QWNK) of murine PrP [102]. This epitope is known to be conserved between mouse and human sequences and corresponds to residues 98–101 of the latter [93], which implies similar interaction between apoE and PrP might take place in human prionoses. It is noteworthy that the 6D11 PrP epitope also was proposed to interact with Aβ oligomers, while its hindrance was shown to prevent binding of Aβ oligomers to excitatory synapses and reduce intraneuronal tau phosphorylation and aggregation [57, 117]. The amount of PrP, which was released from immunoprecipitated complexes under reducing conditions was similar between *ε2/ε2*_22L_, and *ε3/ε3*_22L_ mice but significantly higher in *ε4/ε4*_22L_ animals. This increased ratio between apoE and PrP in *ε4/ε4*_22L_ mice suggests stronger interaction between PrP and apoE4 than between PrP and other apoE isoforms and might explain the propensity for increased formation of large order oligomers observed in *ε4/ε4*_22L_ animals. Since no apoE/PrP complexes were detected in NBH-inoculated control, it is likely that disease specific changes in the PrP protein conformation and/or changes in its physicochemical properties constitute prerequisites for the interaction with apoE. Taking it together, we found numerous aspects of PrP proteinopathy that were significantly enhanced in the presence of the *ε4* allele including elevated levels of the total PrP, PrP^Sc^, detergent insoluble PrP, enhanced PrP oligomerization and evidence of increased complexing of pathologically altered PrP with apoE, which constitute one important mechanism, through which the *ε4* allele negatively affects the outcome of prion disease. The isoform-specific interaction between apoE and various disease-specific misfolded proteins is a recognized mechanism through which apoE propagates aggregation and deposition of these proteins. Besides a well-established effect of apoE directly interacting with Aβ and particularly apoE4 promoting Aβ oligomerization and fibrillization [66, 92, 96, 106, 108], there is evidence derived both from transgenic animal and *in vitro* studies apoE4 may directly promote α-synuclein aggregation [31, 36]. In contrast, *in vitro* studies have identified that recombinant as well as lipidated, apoE2 and to a lesser extent apoE3, but not apoE4 form complexes with recombinant human tau [105, 107, 128].

Prion pathology is inherently associated with early and robust inflammatory activation of astrocytes and microglia [3, 4, 15, 18, 42, 77]. In fact, GFAP-reactive astrogliosis was the first neuropathological metric clearly showing a significant increase in presymptomatic *APOE*-TR mice at 15 wpi. We found a strong *APOE* genotype effect on the magnitude of microglia and astrocyte activation both reflected by differences in the load of activated microglia and astrocytes and differences in microglia and astrocyte specific transcript. Characterization of the transcript using a nanoStringTM nCounter^®^ analysis showed significant upregulation in a number of microglia specific genes, canonically categorized as markers of microglia activation (*Aif1, Csf1r, Cst7, and Siglech*), genes involved in immune response (*C1qa, C1qb, C1qc, C4a/b, C3ar1, Csf3r, Csf1*, and *Ccl3*), and those encoding various microglia recognition receptors (*Axl, Cx3cr1, Fcrls, Clec7a, Mertk, P2ry6, Stab1*, *Trem2*, and *Tyrobp*). Similarly, several categories of astrocyte specific genes were upregulated including reactive astrocyte markers (*Aldh1l1, Aqp4, Gfap, Serpina3n, Slc1a3, Sox9, Vim*), genes involved in antigen presenting and processing (*H2-D1, H2-T23, Tap1*), genes involved in immune response (*Ccl12, Cd14*), and those encoding astrocytic markers, which expression is induced by interferons (*Stat1, Stat2, Stat3, Gbp2, Psmb8*). Nearly all these genes were expressed in *ε4/ε4*_22L_ mice at a significantly higher level than in *ε2/ε2*_22L_, and *ε3/ε3*_22L_ mice, while majority of them also showed significantly higher expression in *ε2/ε2*_22L_ mice compared with *ε3/ε3*_22L_ animals. This *ε4 > ε2 > ε3* allele gradient effect could be demonstrated both through cluster analysis of microglia and astrocyte specific gene sets and comparison of individual gene expression through one-way ANOVA. Among significantly upregulated microglia genes we found *P2ry12*, and *Tmem119*, which together with *Cx3cr1* encoding fractalkine receptor are canonically categorized as microglia homeostatic (M0) genes. Their expression is controlled by TGFβ signaling [13]] and they become commonly downregulated in microglia adopting DAM or MGnD states [14, 39, 49, 53, 84]. However, there also is prior evidence for modest increase in *P2ry12, Tmem119* and *Cx3cr1* transcript in mouse prion models, especially in bulk RNA transcript analysis, what suggests a disease-specific effect on their expression [11, 42, 55, 77].

Several genes were found to be expressed more than 10-folds higher in prion infected mice compared to NBH controls in at least one *APOE* genotype. This list both includes genes specific for microglia *Cst7, C4a/b, Ccl3*, and *Clec7a* and for astrocytes *Gfap, Serpina3n, Vim*, and *Ccl12. Cst7* encodes cystatin F, which is an endosomal cysteine protease inhibitor, and its upregulation has been confirmed across several prion and AD studies most likely as a function of ongoing lysosomal pathology [29, 43, 74, 77]. *C4a/b* encodes isotypes of the complement component C4 and *Ccl3* encodes macrophage inflammatory protein 1α, which both are critically involved in mounting the inflammatory cascade initiated by MGnD [15, 73]. Upregulation of *Clec7a* is a hallmark of adopting by microglia DAM or MGnD reactive state [14, 49, 53] and the gene encodes Dectin-1 representing the C-type lectin receptor involved in the immune system’s recognition and acting as the phagocytosis regulator. Its inhibition was found to attenuate neurodegeneration and excessive synapse elimination by MGnD in P301S tau mutant mice [123]. *Gfap* and *Vim* encode intermediate filament proteins of the astrocyte cytoskeleton, and their upregulation is recognized as universal marker of astrocytic activation [52]. *Serpina3n* encodes Serpin 3 protein (a.k.a. α1-antichymotrypsin), which functions as serine peptidase inhibitor during complement cascade activation, apoptosis and inflammation and its expression is particularly increased in response to IL-1, TNF, and IL-6. Upregulation of *Serpina3n* has been documented both in transmissible prion mouse models and AD transgenic mice and it is closely linked to chronic inflammatory response featured by these models [125]. *Ccl12* encodes CC motif chemokine ligand 12, also known as monocyte chemotactic protein 5 (MCP-5), a small protein, which plays a role in recruiting peripheral immune cells to the site of damage and inflammation and its upregulation previously has been shown in prion disease [19].

Stimulation between chronically reactive microglia and astrocytes in neurodegeneration is bidirectional [82]. To ascertain the effect of *APOE* genotype on this process we used RT-qPCR to quantify expression of *Il1α*, *Tnfα*, *C1qa* encoding respective cytokines IL1α, TNFα and C1QA, which are secreted by MGnD microglia and stimulate acquisition of the chronic reactive state by astrocytes and the *C3* gene encoding complement component 3 protein (C3), which is expressed by reactive astrocytes and reciprocally advances MGnD phenotype [42, 61, 62]. We also used RT-qPCR to quantify expression of specific MGnD markers *Aif1* encoding IBA1, *C3ar1* encoding C3 specific receptor, and *Cx3cr1*. Reactive astrocyte markers included *Gfap*, *Ccl12*, and *Ccl2*, which encodes CC motif chemokine ligand 2. Both CC motif chemokine ligands 12 and 2 are astrocytic secretans that can attract immune cells like microglia to the site of chronic inflammation [50]. Propensity of astrocytes to secrete chemotactic molecules like CC2 and CC12 and proinflammatory factors like C3 suggest they may not only passively contribute to neuroinflammation but rather function as effector cells performing classical innate immune functions and driving the neuroinflammatory cascade [23, 89]. These proinflammatory functions of astrocytes appear to play a particularly important role in prionoses, where paradoxical exacerbation of pathology was observed in microglia deficient mice as it was driven by uninhibited proinflammatory response of astrocytes [11]. In line with the concept of astrocyte-driving neurodegeneration selective removal of astrocytic apoE4 was found to protect against tau mediated neurodegeneration in P301S tau mutant mice [121]. All genes interrogated using RT-qPCR were significantly upregulated in 22L inoculated mice at 23 wpi. but not at 15 wpi. Their transcript level was the highest in *ε4/ε4*_22L_ mice followed by *ε2/ε2*_22L_ and *ε3/ε3*_22L_ animals. Thus, using various transcriptomic approaches we found the strongest proinflammatory effect and evidence for microglia-astrocyte co-stimulatory activation in the setting of the *ε4* allele and to a lesser extent in the setting of the *ε2* allele compared to the *ε3* allele, where the inflammatory response was the least pronounced. This increased neuroinflammatory response associated with the *ε2* allele is most likely responsible for reduced disease latency, accelerated tempo of symptoms progression and increased burden of pathology observed in *ε2/ε2*_22L_ mice compared to *ε3/ε3*_22L_ animals.

The *APOE* polymorphism influences immune response both systemically and within the CNS owing it to the expression of apoE in multiple myeloid-lineage cells, including macrophages, dendritic cells, and microglia [5, 64, 80]. Consistently with the main finding of this study, the *ε4* allele has been generally acknowledged as associated with the strongest inflammatory response, while the immunoregulatory properties of the *ε2* allele have been reported with variable results depending on cell type and inflammatory stimulus. In respect to the systemic response, both *ε2* and *ε4* alleles were found to produce stronger inflammatory effect compared to the *ε3* allele [45, 54, 114]. In respect to CNS-specific studies, intraventricular injection of lipopolysaccharide (LPS) into *APOE*-TR mice [129], or *in vitro* stimulation of microglia isolated from these animals using LPS [69] yielded aggravated and attenuated response in the context to *ε4* and *ε2* alleles compared to the *ε3* allele, respectively. In stark contrast, *in vitro* LPS challenge of astrocytes isolates from *APOE*-TR mice produced the highest release of proinflammatory cytokines and upregulation of nuclear factor-kappa B subunit expression in the context of the *ε2* allele [68]. Interestingly a recent study examining how *APOE* genotype modulates cell-type-specific transcriptomic changes in AD brains revealed that *ε4* carriers feature the strongest upregulation of microglia specific genes and most of pro-inflammatory pathways, it also found *ε2* carriers exhibit strong inflammatory response especially involving IL-6 and IL-1β pathways, which suggests a role of both alleles in promoting inflammatory response in the context of AD pathology [59].

Several bi-transgenic mouse models were generated based on *APOE*-TR mice to directly study the effect of the *APOE* polymorphism on CNS pathology induced by accumulation of disease specific misfolded proteins. In models of Aβ deposition, Aβ plaque load clearly was modified in the rank order of *ε4* > > *ε3* > *ε2* [75, 90], with *ε4* mice featuring the strongest peri-plaque proinflammatory microglia activation [90]. Interestingly modeling of tau pathology has been reported with variable results depending on the model used. Crossing of P301S tau mutant mice [47] with *APOE-TR* mice exacerbated tau accumulation and produced the strongest tau-associated inflammatory response in *ε4* mice, while both the tau pathology load and innate immune response in *ε2* and *ε3* mice were comparable [99]. In contrast, following adenoassociated virus delivery of the P301L tau mutant into the lateral ventricle of *APOE*-TR mice the greatest accumulation of pathology was found in *ε2* mice, while *ε4* allele showed a protective effect compared to *ε3* mice [128]. These variable outcomes could be explained by differences in neuroinflammatory response, which in the P301S model is inherently upregulated, and it was further exacerbated by the presence of the *ε4* allele [47], differences in the size of expressed human tau protein, and specific mutations used in different models, and possibly by existence of direct interaction between tau and apoE isoforms postulated in the P301L tau model [128]. Crossing of A53T α-synucleinopathy model mice onto the *APOE*-TR lines, exacerbated behavioral and pathological metrics in animals expressing the *ε4* allele, while the *ε2* allele attenuated α-synuclein pathology [31]. It is noteworthy, that in this model transcriptomic markers of microglia and astrocyte activation showed no differences across animals expressing various *APOE* alleles. Study using a *Cx3cr1*^*GFP/GFP*^ mouse model of macular degeneration showed exacerbation of the pathology readouts including subretinal inflammatory response in the setting of the *ε2* allele and a protective effect of the *ε4* allele compared to the *ε3* allele [58]. In summary, *APOE*-TR mouse studies have demonstrated both *ε2* and *ε4* allele can exacerbate neurodegeneration in a pathology specific context.

The effect of *APOE* polymorphism on various neurodegenerative diseases also was investigated through a number epidemiological and neuropathological studies carried out in affected patients. Presence of the *ε4* allele has been invariably found to elevate occurrence of LOAD, which risk is 3–4-fold and 12–15-fold increased among carries of a single and two *ε4* copies, compared to *ε3* homozygotes, respectively [25]. In contrast, the *ε2* allele strongly protects against LOAD [24] and the likelihood of LOAD among *ε2* homozygotes was found to be exceptionally low [88]. Irrespective of its effect on increasing the risk of LOAD occurrence, the *ε4* allele in allele dose-dependent manner accelerates the tempo of cognitive decline, brain atrophy, and accumulation of neurofibrillary tangle (NFT) pathology in patients with established disease [1, 21, 99, 112, 113]. Information on how the *ε2* allele may affect progression of established LOAD is limited due to low number of affected *ε2* carriers who also do not carry the *ε4* allele; however, available data suggest NFT pathology load in *ε2/ε3* patients is reduced compared to *ε3/ε3* and *ε3/ε4* individuals [124]. The *ε4* allele also has been associated with a greater severity of Lewy body pathology when controlling for co-associated AD pathology [34]. In contrast, several studies have found *ε2* carriers and in particular *ε2/ε2* homozygotes to present with increased risk and elevated pathology load in primary tauopathies, including progressive-supranuclear palsy [122, 128], corticobasal degeneration [128] and very late onset NFT-predominant dementia [46]. Likewise, the *ε2* allele was implicated as a potential risk factor in aged-related macular degeneration [111]. While in prion diseases epidemiological studies showed no clear effect of *APOE* polymorphism on the risk of disease occurrence [72, 95, 126], clinical and neuropathological studies evaluating possible effects of *APOE* polymorphism on the rate of prion disease progression and pathology burden have not been done due to limited number of available cases, disease diversity, and restrictions related to infection precaution concerning work with human prion material [33, 79, 126]. Therefore, we examined the effect of *APOE* polymorphism on prion pathology using *APOE*-TR mice we infected with 22L mouse adapted scrapie strain and while we found no effect of the *APOE* genotype on extra-CNS PrP^Sc^ accumulation and neuroinvasion, the ensuing brain pathology was significantly intensified in the presence of the *ε4* allele and to lesser extent in the presence of the *ε2* allele.

## Conclusions

**Figure 10:**
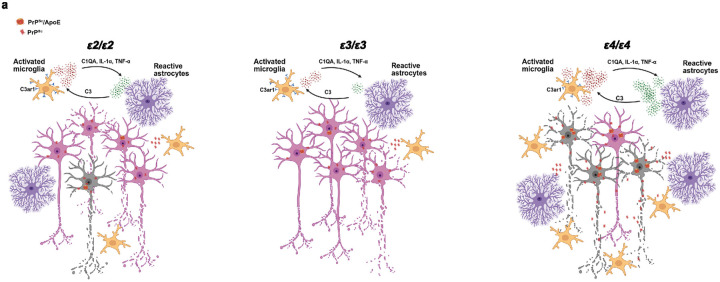
Schematic presentation of the *APOE* genotype effect in prion pathogenesis. In prion disease PrP^C^ undergoes a series of conformational changes, which are associated with reduced solubility, increased oligomerization, and resistance to proteolytic degradation. The ensuing PrP^Sc^ conformer accumulates and causes neuronal demise. Both PrP^Sc^ and neuronal remnants activate microglia, which through secretion of C1QA, IL1a, and TNFa activate astrocytes. In turn, activated astrocytes secrete complement component 3 (C3), which reciprocally stimulate microglia through the C3ar1 receptor. This self-propagating microglia-astrocyte pro-inflammatory feedback loop is an effective driver of neuroinflammation. The *APOE e4* allele produces a double hit effect by fostering disease specific changes in the PrP protein and increasing its accumulation and by promoting the microglia-astrocyte inflammatory response. The *e2* allele is associated with a single hit effect. It does not influence disease specific changes in the PrP protein but promotes inflammatory response compared to the *e3* allele. Created in BioRender. Lizinczyk, A.M. (2025) https://BioRender.com/2vhco40

Findings of our study indicate that *APOE* polymorphism differentially regulates the progression of prion pathology. We identified two mechanisms attributable to detrimental effect endowed by the *ε4* allele, which are increased conversion and accumulation of the PrP^Sc^ conformer and worsening of prion-associated neuroinflammation, while the *ε2* allele was found to be associated with increased inflammatory response. Our findings suggest both *ε4* and *ε2* alleles are disadvantageous determinants in prion pathology (Fig. 10).

## Supplementary Material

Supplementary Files

This is a list of supplementary files associated with this preprint. Click to download.

- SupplementaryFile1LizinczyAMetal.pdf
- SupplementaryFile2LizinczykAMetal.pdf
- SupplementaryFile3LizinczykAMetal.pdf

## Figures and Tables

**Table 1:** List of antibodies used for Western immunoblotting

Antigen	Host	Dilution	Source	Cat. #	Clone
β-actin	Mouse	1:10,000	Sigma- Aldrich, St. Louis, MO	A2228	AC-74
Cluster of differentiation (CD) 230*	Mouse	1:5,000**	BioLegend, San Diego, CA	808001	6D11
Glial fibrillary acidic protein (GFAP)	Rabbit	1:15,000	Dako/Agilent Technologies, Santa Clara, CA	Z0334	
Human apoE	Goat	1:5,000	Meridian Life Science Inc., Memphis, TN	K74180B	
HRP-linked anti-goat IgG	Donkey	1:25,000	Santa Crus Biotechnology Inc., Dallas, Tx	Sc-2020	
HRP-linked anti-rabbit IgG F(ab’)2 specific	Donkey	1:30,000	Cytiva, Marlborough, MA	NA9340	
HRP-linked anti-mouse IgG F(ab’)2 specific	Sheep	1:30,000	Cytiva, Marlborough, MA	NA9310	

* CD230 denotes prion protein (PrP)

** for Western immunoblot detection of immunoprecipitated PrP, dilution of 6D11 was increased to 1:2,000

**Table 2:** List of antibodies used for immunohistochemistry

Antigen	Host	Dilution	Source	Cat. #	Clone
Cluster of differentiation (CD) 68	Rat	1:250	Abcam Inc., Cambridge, MA	Ab53444	FA-11
Cluster of differentiation (CD) 230*	Mouse	1:200	BioLegend, San Diego, CA	808001	6D11
Complement component 3 (C3)	Rat	1:100	Hycult Biotech, Uden, Netherlands	HM1045	11H9
Glial fibrillary acidic protein (GFAP)	Rabbit	1:2,000	Dako/Agilent Technologies, Santa Clara, CA	Z0334	
Ionized calcium adaptor protein 1 (IBA1)	Rabbit	1:1,000	Wako Chemicals Inc., Richmond, VA	019-19741	
Alexa 594-conjugated anti-mouse IgG	Goat	1:500	Jackson Immuno Research Labs, West Grove, PA	115-585-146	
Alexa 488- conjugated anti-rabbit IgG	Goat	1:500	Jackson Immuno Research Labs	111-545-144	
Alexa 594- conjugated anti-rabbit IgG	Goat	1:500	Jackson Immuno Research Labs	111-585-144	
Alexa 594- conjugated anti-rat IgG	Goat	1:500	Jackson Immuno Research Labs	112-585-143	

* CD230 denotes prion protein (PrP)

**Table 3:** List of primer sequences used for RT-qPCR

Gene Symbol	Forward Primer Sequence (5’ – 3’)	Reverse Primer Sequence (5’ – 3’)
*Gapdh*	AGGTCGGTGTGAACGGATTTG	TGTAGACCATGTAGTTGAGGTCA
*C3*	CCAGCTCCCCATTAGCTCTG	GCACTTGCCTCTTTAGGAAGTC
*Il1α*	CGCTTGAGTCGGCAAAGAAAT	CTTCCCGTTGCTTGACGTTG
*C1qa*	AAAGGCAATCCAGGCAATATCA	TGGTTCTGGTATGGACTCTCC
*Aif*	TCTTCGTTTTACCATCAGCC	TGACACGGACGCTGGCCTGAA
*C3ar1*	TCGATGCTGACACCAATTCAA	TCCCAATAGACAAGTGAGACCAA
*Cx3cr1*	CAGCATCGACCGGTACCTT	GCTGCACTGTCCGGTTGTT
*Gfap*	GGCGCTCAATGCTGGCTTCA	TCTGCCTCCAGCCTCAGGTT
*Ccl12*	ATTTCCACATTCTATGCCTCCT	ATCCAGTATGGTCCTGAAGATCA
*Ccl2*	CACTCACCTGCTGCTACTCA	GCTTGGTGACAAAAACTACAGC
*Tnfα*	TGTGCTCAGAGCTTTCAACAA	CTTGATGGTGGTGCATGAGA

## Data Availability

Raw images and datasets generated and analyzed during the current study are available from the corresponding author upon a reasonable request. In addition, NanoString nCounter^®^ datasets of microglia and astrocyte transcript can be accessed through the Gene Expression Omnibus Repository, [https://www.ncbi.nlm.nih.gov/geo/query/acc.cgi?acc=GSE307182] (https:/www.ncbi.nlm.nih.gov/geo/query/acc.cgi?acc=GSE307182).

## Abbreviations

Ab: b-amyloid
ANOVA: analysis of variance
apoE: apolipoprotein E protein
Apoe: apoE gene (murine)
APOE: apoE gene (human)
BCA: bicinchoninic acid
C3: complement component 3
CD68: cluster of differentiation 68
CD230: cluster of differentiation 230 (a.k.a PrP)
CJD: Creutzfeldt-Jakob disease
CNS: central nervous system
DAM: disease associated microglia
DPBS: Dulbecco’s phosphate buffered saline
dpi: days post inoculation
GFAP: glial fibrillary acidic protein
Iba1: Ionized calcium binding adaptor molecule
ID: integrated density
IFN: interferon
LOAD: late onset Alzheimer’s disease
LPS: lipopolysaccharide
LRS: lymphoreticular system
M1: primary motor cortex
MGnD: microglial neurodegenerative phenotype
NaPTA: sodium phosphotungustic acid
NBH: normal brain homogenate
NFT: neurofibrillary tangle
OD: optical density
PK: proteinase K
LRS: lymphoreticular system
PrP: prion protein
PrPSc: scrapieform conformer of prion protein
RT-qPCR: Reverse Transcription Quantitative Polymerase Chain Reaction
S1: primary somatosensory cortex
SEM: standard error
SDS-PAGE: sodium dodecyl sulfate-polyacrylamide gel electrophoresis
THB: tissue homogenization buffer
TR: target replacement
TSS: Total Scrapie Score
wpi: weeks post inoculation
